# Postprandial exercise regulates tissue-specific triglyceride uptake through angiopoietin-like proteins

**DOI:** 10.1172/jci.insight.181553

**Published:** 2024-08-22

**Authors:** Xiaomin Liu, Yiliang Zhang, Bingqian Han, Lin Li, Ying Li, Yifan Ma, Shijia Kang, Quan Li, Lingkai Kong, Kun Huang, Bao-liang Song, Yong Liu, Yan Wang

**Affiliations:** 1 Hubei Key Laboratory of Cell Homeostasis, College of Life Sciences, TaiKang Center for Life and Medical Sciences, Wuhan University, Wuhan, China.; 2 Tongji School of Pharmacy, Huazhong University of Science and Technology, Wuhan, China.

**Keywords:** Metabolism, Homeostasis, Insulin, Lipoproteins

## Abstract

Fuel substrate switching between carbohydrates and fat is essential for maintaining metabolic homeostasis. During aerobic exercise, the predominant energy source gradually shifts from carbohydrates to fat. While it is well known that exercise mobilizes fat storage from adipose tissues, it remains largely obscure how circulating lipids are distributed tissue-specifically according to distinct energy requirements. Here, we demonstrate that aerobic exercise is linked to nutrient availability to regulate tissue-specific activities of lipoprotein lipase (LPL), the key enzyme catabolizing circulating triglyceride (TG) for tissue uptake, through the differential actions of angiopoietin-like (ANGPTL) proteins. Exercise reduced the tissue binding of ANGPTL3 protein, increasing LPL activity and TG uptake in the heart and skeletal muscle in the postprandial state specifically. Mechanistically, exercise suppressed insulin secretion, attenuating hepatic *Angptl8* transcription through the PI3K/mTOR/CEBPα pathway, which is imperative for the tissue binding of its partner ANGPTL3. Constitutive expression of ANGPTL8 hampered lipid utilization and resulted in cardiac dysfunction in response to exercise. Conversely, exercise promoted the expression of ANGPTL4 in white adipose tissues, overriding the regulatory actions of ANGPTL8/ANGPTL3 in suppressing adipose LPL activity, thereby diverting circulating TG away from storage. Collectively, our findings show an overlooked bifurcated ANGPTL-LPL network that orchestrates fuel switching in response to aerobic exercise.

## Abstract 2

Exercise coordinates with feeding and regulates tissue specific fuel switching through bifurcated regulation of Angiopoietin-like (ANGPTL) proteins

## Graphical abstract

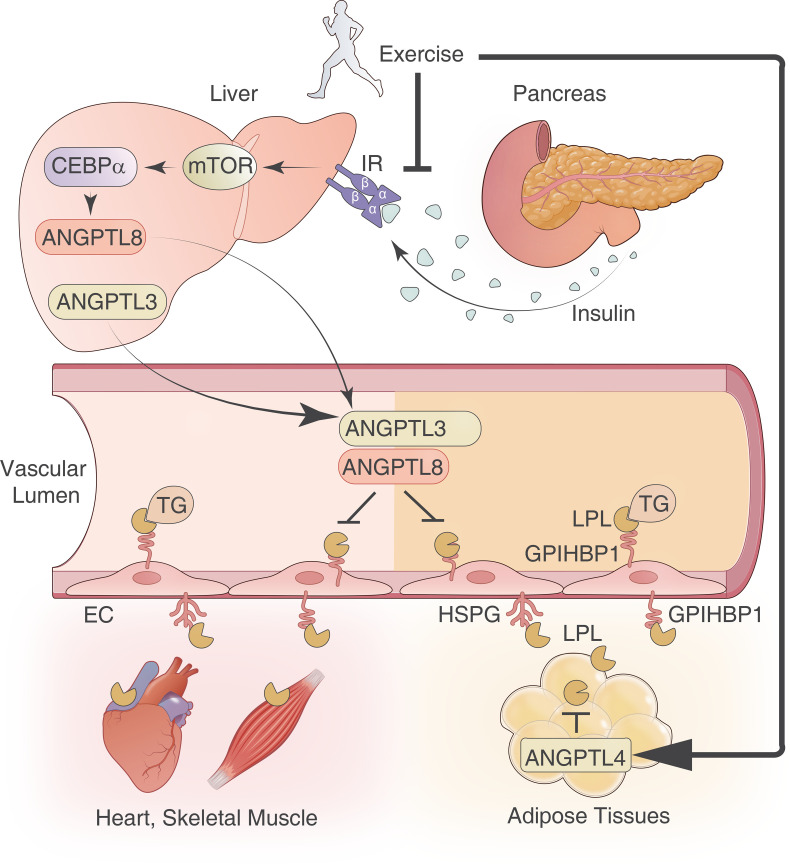

## Introduction

Carbohydrates and fat are the predominant energy substrates in mammals. Extensive studies have demonstrated the utilization and switching of substrates between carbohydrates and fats during exercise (1). This substrate switching is crucial for maintaining blood glucose levels and sustaining exercise capacity (2).

Circulating glucose, nonesterified free fatty acids (FFAs), and triglycerides (TGs) are 3 major metabolic fuels that empower the heart and skeletal muscle for energy production or are taken up by white adipose tissues (WATs) for storage. Glucose and FFA are taken up by tissues primarily through transporter-mediated mechanisms, whose activities are regulated by exercise through multiple pathways (3, 4). Tissue TG uptake is tightly controlled by lipoprotein lipase (LPL), a master TG hydrolysis lipase that sits on the capillary lumen of the endothelial surface (5–8).

The heart prefers fatty acids over carbohydrates for energy production (9–12). Among total fatty acids taken up by the heart, about 60% in humans, or as much as 80% in dogs, are derived from circulating TGs, with the remainder derived from FFAs (13). The essential role of TG-derived fatty acids has been elucidated by the heart-specific LPL-knockout mice (14, 15). However, whether and how exercise regulates tissue LPL activity and TG-derived fatty acid uptake, especially in tissue-specific manner, remains obscure (16–19).

The angiopoietin-like (ANGPTL) family comprises 8 secreted proteins (20). Among the family members, mutations in *ANGPTL3*, *ANGPTL4*, and *ANGPTL8* are associated with dyslipidemia (20, 21). ANGPTL3 is dominantly expressed in the liver (22). ANGPTL4 and ANGPTL8 are mainly expressed in both the liver and adipose tissues (20, 23–25). All 3 members, ANGPTL3, ANGPTL4, and ANGPTL8, share a conserved LPL binding motif and act as LPL inhibitors (20). Recently, we showed that feeding increases the expression and hepatic secretion of ANGPTL8, which forms a complex with ANGPTL3 and inhibits peripheral tissue LPL activity in the heart, skeletal muscle, and WAT postprandially (20, 26, 27). In contrast, ANGPTL4 is a fasting-induced factor and is reported to inhibit LPL activity in adipose tissue locally (28–31). Individuals with ANGPTL3 deficiency exhibit combined hypolipidemia, and an ANGPTL3 inhibitory antibody has been approved for treating homozygous familial hypercholesterolemia (32, 33). A previous study reported that exercise increases ANGPTL4 expression in the resting muscle and diverts circulating TG to the exercising muscle for energy production (34). However, whether and how ANGPTL3 and ANGPTL8 regulate the lipid metabolic response to exercise remains unknown.

In the present study, we found that aerobic exercise is coupled to nutrient availability and increases postprandial LPL activity and TG-derived fatty acid uptake in the heart and skeletal muscle. Mechanistically, exercise suppressed insulin secretion, decreasing ANGPTL3 tissue binding and reducing its inhibition for peripheral tissue LPL activity via a reduction of ANGPTL8 expression through the hepatic PI3K/mTOR/CEBPα signaling pathway. In contrast, exercise increased the expression of ANGPTL4, which overrides the regulatory action of the ANGPTL8/ANGPTL3 complex and suppresses LPL activity and TG-derived fatty acid uptake in WATs to divert circulating TG away from storage. Thus, our findings reveal a bifurcated ANGPTL-LPL network that orchestrates fuel switching through tissue-specific utilization of TG-derived fatty acids in response to aerobic exercise.

## Results

### Exercise suppression of ANGPTL3 tissue binding increases LPL activity and promotes TG uptake in energy-consumption tissues in the postprandial stage.

**Figure 1:**
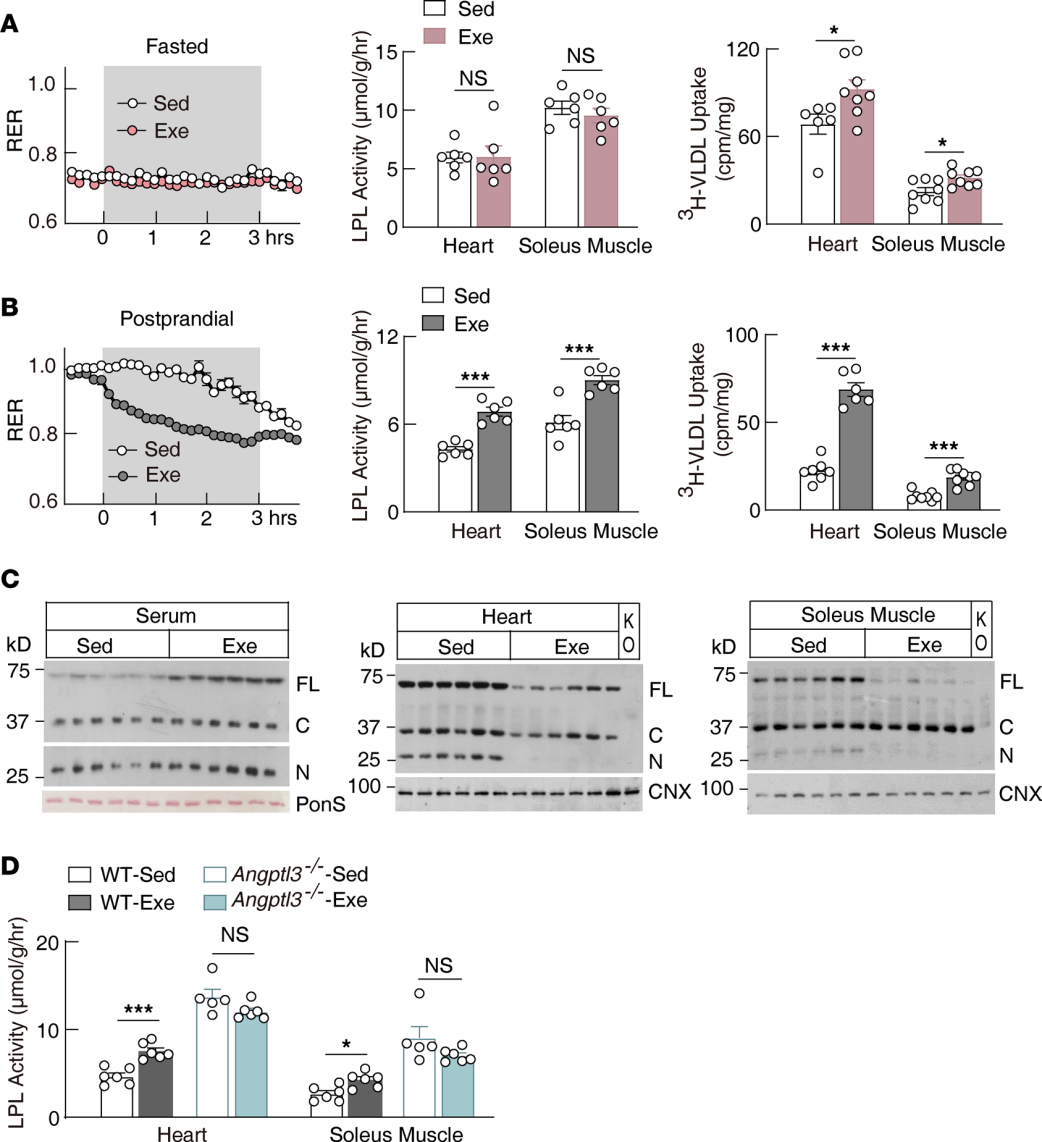
Exercise suppression of ANGPTL3 tissue binding increases LPL activity in the heart and skeletal muscle in the postprandial state. (**A**) Respiratory exchange ratio (RER), tissue LPL activity, and triolein-[^3^H]palmitate uptake in mice exercised at fasting (*n* = 12 males/group for RER, *n* = 6 males/group for tissue LPL activity and triolein uptake, 8–10 weeks of age). (**B**) Respiratory exchange ratio, tissue LPL activity, and triolein-[^3^H]palmitate uptake in mice exercised postprandially (*n* = 12 males/group for RER, *n* = 6 males/group for tissue LPL activity and triolein uptake, 8–10 weeks of age). (**C**) ANGPTL3 protein levels in the serum, heart, and soleus muscle of mice following postprandial exercise as in **B** (*n* = 6 males/group, 8–10 weeks of age). (**D**) Tissue LPL activity in *Angptl3*^–/–^ mice and littermate control wild-type mice following postprandial exercise as in **B** (*n* = 5–6 males/group, 13–17 weeks of age). Sed, sedentary; Exe, exercise; FL, ANGPTL3 full-length; C, ANGPTL3 C-terminal; N, ANGPTL3 N-terminal; PonS, ponceau S; CNX, calnexin; KO, samples from *Angptl3*^–/–^ mice. All experiments were repeated with similar results. Data are shown as the mean ± SEM. *P < 0.05, ***P < 0.001.

Fuel utilization is modulated by both physical activity and feeding status (35). We first characterized the metabolic changes during a single bout of aerobic exercise, either at the fasting or postprandial state (Supplemental Figure 1, A and B; supplemental material available online with this article; https://doi.org/10.1172/jci.insight.181553DS1). Exercise markedly increased the metabolic rate in both fasting and postprandial conditions (Supplemental Figure 1, A and B). However, exercise specifically promoted the fuel switching during the postprandial period (Figure 1, A and B). Concurrently, postprandial exercise significantly increased both LPL activity and TG-derived fatty acid uptake in energy-consumption tissues; we used the heart and soleus muscle as two representative tissues (Figure 1B).

Fat is preferred over carbohydrates in aerobic exercise (36–41). Consistent with this, low-to-moderate intensity exercises all elicited similar changes in tissue LPL activity (Supplemental Figure 2, A and B), while high-intensity exercise did not have the ability to regulate soleus muscle LPL activity (Supplemental Figure 2C), which aligns with the preference for carbohydrates over fat during anaerobic exercise. We selected the postprandial moderate-intensity exercise (14 m/min, 3 hours) for further mechanistic study.

ANGPTL3 is a hepatokine that inhibits peripheral tissue LPL activity through unknown mechanisms (26). It is cleaved at a proprotein convertase consensus site, and it was considered an activation step during secretion (42). However, subsequent studies suggested that both the N-terminal and full-length forms of ANGPTL3 possess similar potency for LPL inhibition (43). Interestingly, we found that exercise decreased both the full-length and N-terminal forms of ANGPTL3 in the heart and soleus muscle, even though its hepatic transcriptional level or circulating protein level remained unchanged or slightly increased (Figure 1C and Supplemental Figure 2D). Exercise-responsive LPL regulation and TG-derived fatty acid uptake were completely lost in the heart and soleus muscle of *Angptl3*-knockout (*Angptl3^–/–^*) mice (Figure 1D and Supplemental Figure 2E). These findings suggest that exercise upregulates LPL activity and promotes TG-derived fatty acid uptake in energy-consumption tissues by reducing ANGPTL3 tissue binding. However, LPL activity in wild-type mice after exercise was still lower than the activity in sedentary *Angptl3^–/–^* mice, suggesting that exercise does not abolish ANGPTL3 function in tissues completely, which is consistent with the findings that exercise only partially decreases full-length ANGPTL3 in tissues. Skeletal muscle and heart respond to exercise similarly for LPL regulation and TG uptake, we will use the heart as a representative energy-consumption tissue for further mechanistic study.

### ANGPTL8 mediates exercise suppression of ANGPTL3 tissue binding.

**Figure 2:**
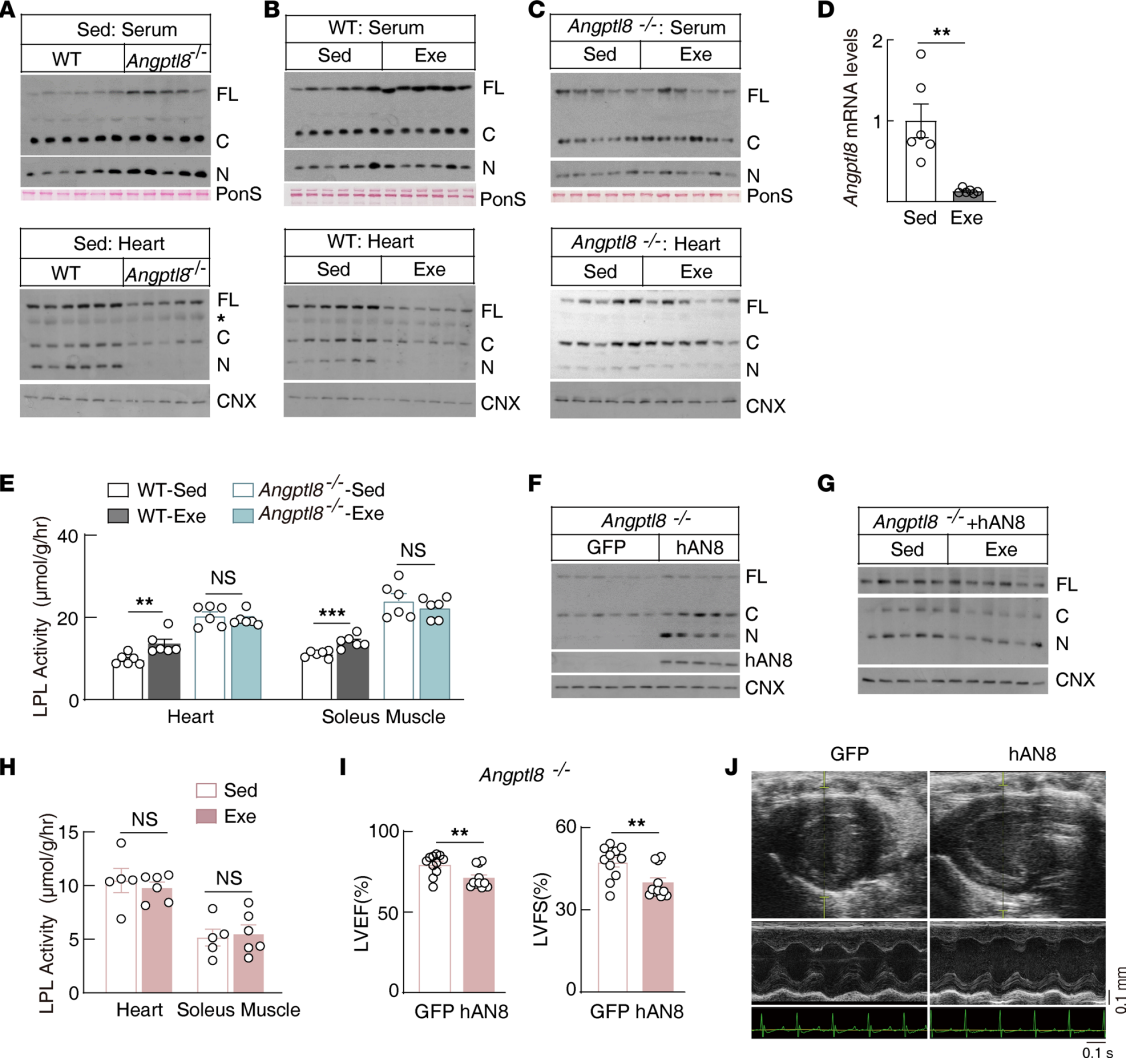
ANGPTL8 mediates exercise suppression of ANGPTL3 tissue binding in the postprandial state. (**A**–**C**) ANGPTL3 protein levels in *Angptl8*-knockout (*Angptl8*^–/–^) mice and littermate control wild-type mice with or without exercise in the postprandial state (*n* = 5–6 females/group, 8–18 weeks of age; the asterisk indicates nonspecific bands). (**D**) Hepatic *Angptl8* mRNA levels of wild-type mice following postprandial exercise (*n* = 6 males/group, 8–10 weeks of age). (**E**) Heart LPL activity of *Angptl8*^–/–^ mice and littermate control wild-type mice following postprandial exercise (*n* = 6 males/group, 9–14 weeks of age). (**F**) Protein levels in the heart of *Angptl8*^–/–^ mice with hepatic specific expressing of GFP or human ANGPTL8 (hAN8) (*n* = 5–6 males/group, 7–8 weeks of age). Human ANGPTL8 or GFP was expressed in mice with adeno-associated virus (AAVs) with the hepatic specific Thyroxine binding globulin (TBG) promoter as described in *Methods*. (**G**) ANGPTL3 protein levels in hearts of *Angptl8*^–/–^ mice with hepatic specific expression of human ANGPTL8 following postprandial exercise (*n* = 5–6 males/group, 7–8 weeks of age). ANGPTL8 expression was performed as in **F**. (**H**) LPL activity in heart and soleus muscle of mice used in **G**. (**I**) Systolic heart function in *Angptl8*^–/–^ mice expressing GFP or human ANGPTL8. GFP- or ANGPTL8-expressing mice were prepared as in **F**, and heart function was evaluated after a 2-week exercise challenge, as described in the Methods (*n* = 12 males/group, 8–11 weeks of age). LVEF, left ventricular ejection fraction; LVFS, left ventricular fractional shortening. (**J**) Representative M-mode images of the echocardiographic analysis for **I**. Scale bar: 0.1 s (*x* axis); 0.1 mm (*y* axis). All experiments were repeated with similar results. Data are shown as the mean ± SEM. **P < 0.01, ***P < 0.001.

Previous studies have demonstrated that ANGPTL3 alone is a poor LPL inhibitor and its activity is greatly enhanced when it forms a complex with ANGPTL8 (20, 43–47). However, the mechanism by which this complex inhibits LPL activity remains unknown. We observed that *Angptl8*-knockout (*Angptl8^–/–^*) mice had normal or slightly increased ANGPTL3 levels in the circulation but had greatly reduced levels in the heart, whereas in *Angptl8^–/–^* mice, exercise was unable to regulate ANGPTL3 tissue binding (Figure 2, A–C). Meanwhile, exercise significantly suppressed *Angptl8* transcription in the liver (Figure 2D), suggesting that exercise regulates tissue ANGPTL3 levels by suppressing *Angptl8* expression. Consistently, exercise was unable to regulate tissue LPL activity and TG-derived fatty acid uptake in *Angptl8^–/–^* mice (Figure 2E and Supplemental Figure 2F).

Reexpressing human ANGPTL8 specifically in the livers of *Angptl8^–/–^* mice rescued ANGPTL3 tissue binding (Figure 2F). This constitutively expressed ANGPTL8 was not regulated by exercise (Supplemental Figure 2G), and exercise failed to regulate ANGPTL3 tissue binding and LPL activity (Figure 2, G and H). These findings strongly suggest that exercise suppresses ANGPTL3 binding and releases its LPL inhibition in energy-consumption tissues through suppressing the hepatic *Angptl8* transcription.

A constant energy supply is essential for normal heart function. *Angptl8^–/–^* mice constitutively expressing human ANGPTL8 had increased glucose uptake in response to exercise (Supplemental Figure 2H). However, these mice displayed cardiac dysfunction after exercise challenge (Figure 2, I and J, and Supplemental Figure 2, I and J). These data suggest that the regulated *Angptl8* transcription is essential for normal heart function in response to exercise.

### Exercise suppresses Angptl8 expression through the hepatic insulin/mTOR/CEBPα signaling pathway.

**Figure 3:**
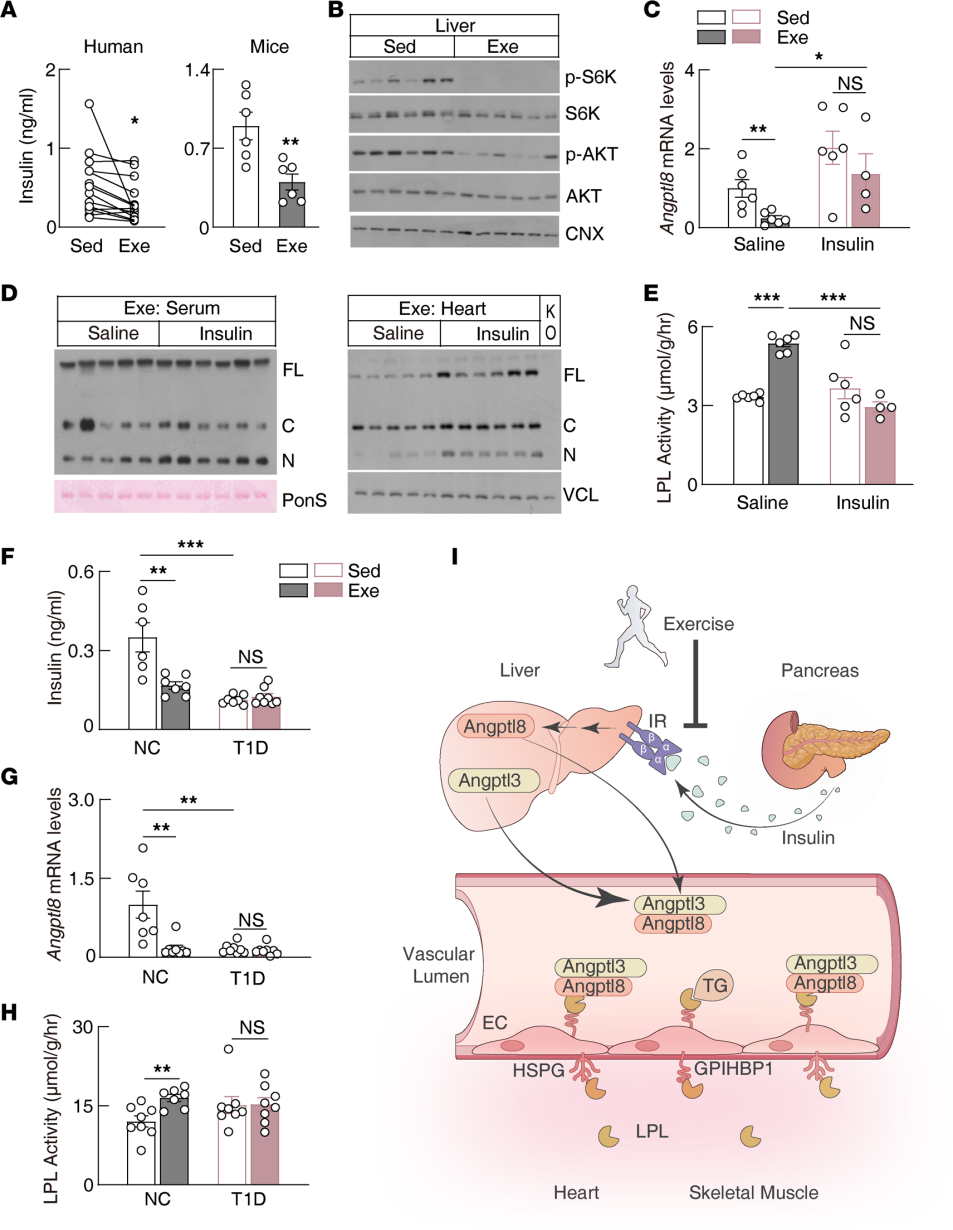
Postprandial exercise suppresses hepatic *Angptl8* expression via reduction of insulin. (**A**) Serum insulin levels in humans and mice following postprandial exercise (humans: 8 male and 6 female individuals; aged 25 **±** 5 years; mice: *n* = 6 males/group, 8–10 weeks of age). (**B**) Immunoblot analysis of liver lysates in mice following postprandial exercise (*n* = 6 males/group, 8–10 weeks of age). (**C**) Hepatic *Angptl8* transcriptional levels in mice exercised postprandially with or without insulin supplementation (*n* = 4–6 males/group, 8–10 weeks of age). (**D**) ANGPTL3 protein levels in mice exercised postprandially with or without insulin supplementation (*n* = 5–6 males/group, 8–10 weeks of age). (**E**) Heart LPL activity in mice used in **C**. (**F**–**H**) Serum insulin levels, hepatic *Angptl8* transcriptional levels, and heart LPL activity in normal (NC) or type 1 diabetic (T1D) mice exercised postprandially (*n* = 8 males/group, 8–10 weeks of age). (**I**) Working model for postprandial exercise upregulation of LPL activity in the heart and skeletal muscle. EC, endothelial cells; IR, insulin receptor; HSPG, heparin sulfate proteoglycans. All experiments were repeated with similar results. Data are shown as the mean ± SEM. *P < 0.05, **P < 0.01, ***P < 0.001.

Transcriptional profiling revealed that the hepatic insulin signaling pathway was most dramatically regulated by exercise (Supplemental Figure 3A). Previous studies showed that insulin increases the expression of *ANGPTL8* and increases the secretion of the ANGPTL8/ANGPTL3 complex (46, 48–51), though the insulin downstream signaling pathway remains obscure (50, 51). We found that exercise reduced insulin levels both in humans and in mice (Figure 3A) and reduced the activity of the hepatic insulin signaling pathway (Figure 3B). Insulin supply reversed hepatic *Angptl8* transcription, rescued heart ANGPTL3 binding, and suppressed heart LPL activity during exercise (Figure 3, C–E). Type 1 diabetic mice have a deficiency in insulin secretion. Consistently, exercise failed to suppress *Angptl8* transcription and failed to increase heart LPL activity in these mice (Figure 3, F–H). These data suggest that the reduction of insulin is the driving force for the upregulation of heart LPL activity in response to exercise (Figure 3I), which is consistent with the finding that exercise failed to decrease heart ANGPTL3 binding (Supplemental Figure 3B) and failed to increase heart LPL activity and TG uptake at fasting state when insulin levels are low (Figure 1A).

**Figure 4:**
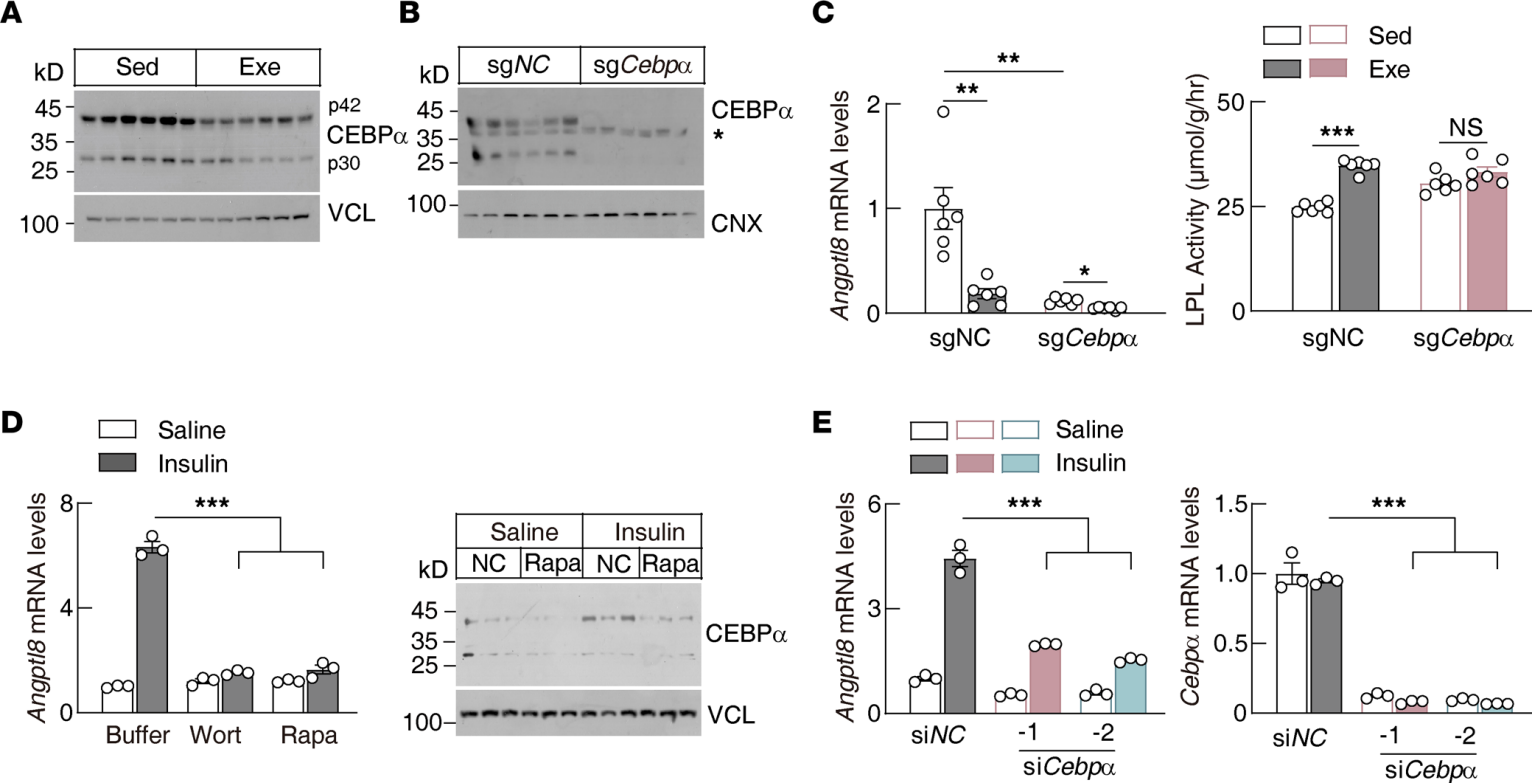
Insulin regulates hepatic *Angptl8* transcription through the PI3K/mTOR/CEBPα pathway. (**A**) Hepatic CEBPα protein levels in mice following postprandial exercise (*n* = 6 males/group, 8–10 weeks of age; p42 and p30 are 2 different isoforms for CEBPα). (**B**) Hepatic CEBPα protein levels in *Cebp*α-knockout mice (*n* = 6 males/group, 8–14 weeks of age; the asterisk indicates nonspecific bands). SpCas9-transgenic mice were infused with AAVs expressing gRNA targeting *Cebp*α (5 × 10^11^ particles/mouse) through the tail vein. Fourteen days later, samples were collected at the sedentary state. A scramble gRNA was used as negative control (sg*NC*). (**C**) Hepatic *Angptl8* transcriptional level and heart LPL activity in *Cebpα*-knockout mice following postprandial exercise. *Cebpα* knockout was performed exactly as in **B**. Fourteen days later, mice were subjected to exercise and samples were analyzed as described in Methods (*n* = 6 males/group, 8–14 weeks of age). (**D**) *Angptl8* transcriptional level and CEBPα protein level in rat primary hepatocytes with indicated treatments (*n* = 3/group). Wort, wortmannin; Rapa, rapamycin. (**E**) Insulin-regulated *Angptl8* transcription in rat primary hepatocytes with or without (si*NC*) *Cebp*α knockdown (*n* = 3/group). All experiments were repeated with similar results. Data are shown as the mean ± SEM. *P < 0.05, **P < 0.01, ***P < 0.001.

ANGPTL8 is also regulated by feeding and is highly conserved between human and mice (20). Bioinformatics analysis revealed 6 conserved transcription factor binding sites in the promoter region of *Angptl8* (Supplemental Figure 3C), among which only *Cebpα* was regulated both by fasting and by exercise (Supplemental Figure 3D). CEBPα is a transcription factor that plays important roles in hepatic lipid metabolism and adipogenesis (52). Chromatin immunoprecipitation and sequencing identified 2 conserved CEBPα binding motifs in the promoter region of *ANGPTL8* (Supplemental Figure 3E). Notably, exercise decreased hepatic CEBPα protein levels, especially for the longer isoform p42 (Figure 4A), which is also the active isoform for transcriptional regulation (52). Hepatic *Cebpα* knockout decreased the hepatic *Angptl8* transcription and abolished heart LPL regulation in response to exercise (Figure 4, B and C).

Insulin increased *Angptl8* transcription in rat primary hepatocytes directly, which was blocked by the phosphoinositide 3-kinase (PI3K) inhibitor (wortmannin) and mechanistic target of rapamycin (mTOR) inhibitor (rapamycin), suggesting that insulin regulated *Angptl8* transcription through the canonical PI3K/mTOR signaling pathway (Figure 4D). Consistently, insulin increased the CEBPα level in primary hepatocytes and was blocked by rapamycin (Figure 4D). Knocking down *Cebpα* suppressed *Angptl8* transcription in primary hepatocytes (Figure 4E). Taken together, these data suggest that exercise suppresses hepatic *Angptl8* transcription, at least partially through the insulin/mTOR/CEBPα signaling pathway.

### Increased ANGPTL4 counteracts the decreased ANGPTL3 for LPL inhibition in response to exercise in WATs.

**Figure 5:**
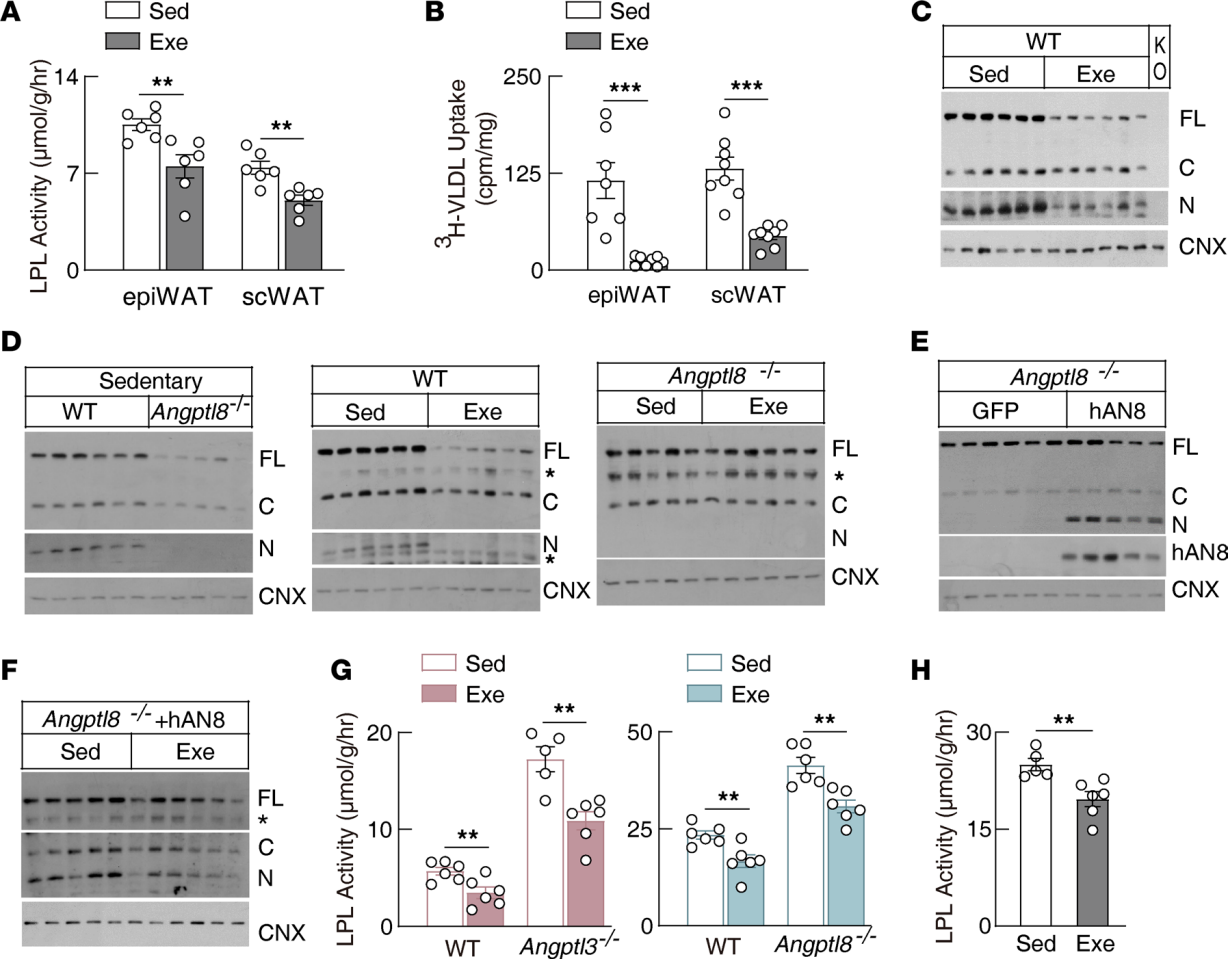
Postprandial exercise suppresses white adipose tissue LPL activity independently of ANGPTL3 or ANGPTL8. (**A** and **B**) Tissue LPL activity and triolein-[^3^H]palmitate uptake in mice following postprandial exercise (*n* = 6 males/group, 8–10 weeks of age). epiWAT, epididymal white adipose tissue; scWAT, subcutaneous white adipose tissue. (**C**) ANGPTL3 protein level in epiWAT of wild-type mice following postprandial exercise (*n* = 6 males/group, 8–10 weeks of age). (**D**) ANGPTL3 protein level in epiWAT of *Angptl8^–/–^* mice and littermate controls following postprandial exercise (*n* = 5–6 females/group, 8–18 weeks of age; the asterisk indicates nonspecific bands). (**E**) Immunoblot of epiWAT in *Angptl8^–/–^* mice with liver-specific expression of GFP or human ANGPTL8 (hAN8) in the postprandial state (*n* = 5–6 males/group, 7–8 weeks of age). (**F**) ANGPTL3 protein level in epiWAT of *Angptl8^–/–^* mice with liver-specific expressing of human ANGPTL8 following postprandial exercise (*n* = 5–6 males/group, 7–8 weeks of age; the asterisk indicates nonspecific bands). (**G**) LPL activity in epiWAT of *Angptl3^–/–^* mice, *Angptl8^–/–^* mice, and their wild-type littermate controls following postprandial exercise (*Angptl3^–/–^*: *n* = 5–6 males/group, 13–17 weeks of age; *Angptl8^–/–^*: *n* = 6 males/group, 9–14 weeks of age). (**H**) LPL activity in epiWAT of mice used in **F**. All experiments were repeated with similar results. Data are shown as the mean ± SEM. **P < 0.01, ***P < 0.001.

WAT is the major organ for TG storage. We found that exercise significantly suppressed LPL activity and TG-derived fatty acid uptake in WAT, accompanied by a decreased ANGPTL3 tissue binding (Figure 5, A–C). Similar to the heart (Figure 2), exercise decreased WAT ANGPTL3 binding through the suppression of hepatic *Angptl8* transcription (Figure 5, D–F). However, exercise-suppressed LPL activity was largely preserved in WAT of *Angptl3^–/–^* and *Angptl8^–/–^* mice (Figure 5G) as well as in the mice with constitutive expression of human ANGPTL8 (Figure 5H). Taken together, these data suggest that exercise suppresses WAT LPL activity independent of ANGPTL3 or ANGPTL8.

**Figure 6:**
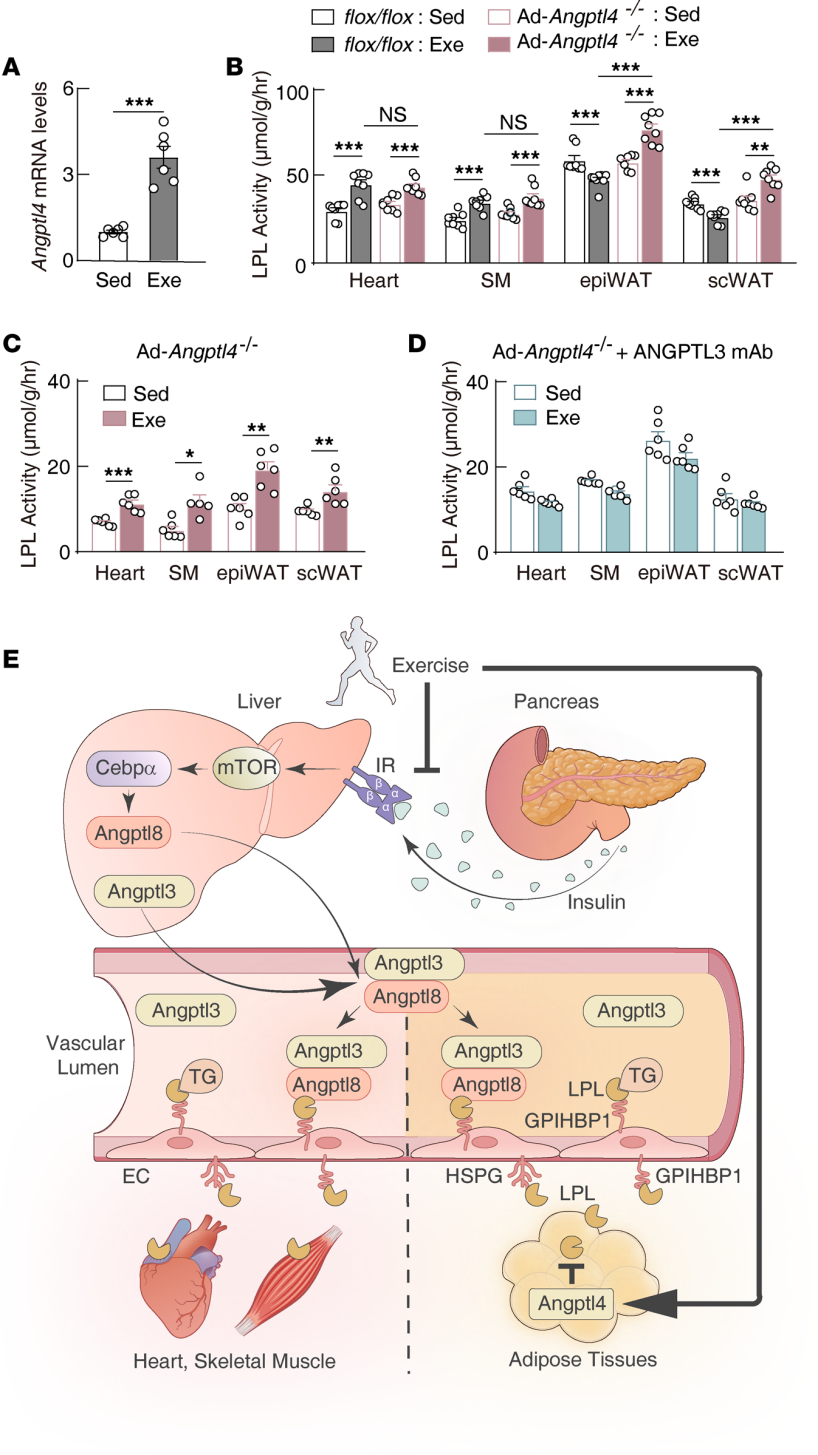
Postprandial exercise upregulation of local *Angptl4* expression predominantly suppresses white adipose tissue LPL activity. (**A**) A*ngptl4* transcriptional level in epiWAT of mice following postprandial exercise (*n* = 6 males/group, 8–10 weeks of age). (**B**) Tissue LPL activity in adipose tissue–specific *Angptl4*-knockout (Ad-*Angptl4^–/–^*) mice and littermate control wild-type mice following postprandial exercise (*n* = 8 males/group, 7–12 weeks of age). (**C**) Tissue LPL activity in Ad-*Angptl4^–/–^* mice following postprandial exercise (*n* = 6 males/group, 7–10 weeks of age). (**D**) Tissue LPL activity in Ad-*Angptl4^–/–^* mice treated with ANGPTL3 inhibitory antibody and exercised postprandially (*n* = 6 females/group, 8–11W). ANGPTL3 inhibitory antibody (mAb) was infused through the tail vein (20 mg/kg), and postprandial exercise was performed at 4 days later. SM, soleus muscle; epiWAT, epididymal white adipose tissue; scWAT, subcutaneous white adipose tissue. All experiments were repeated with similar results. Data are shown as the mean ± SEM. *P < 0.05, **P < 0.01, ***P < 0.001. (**E**) Working model for postprandial exercise regulation of tissue-specific LPL activity and TG-derived fatty acid uptake. Exercise increases LPL activity and TG-derived fatty acid uptake in the heart and skeletal muscle through suppression of the hepatic insulin/mTOR/CEBPα/ANGPTL8/ANGPTL3 axis, while exercise suppresses adipose tissue LPL activity and TG-derived fatty acid uptake through upregulation of ANGPTL4. EC, endothelial cells; IR, insulin receptor; HSPG, heparin sulfate proteoglycans.

ANGPTL4 is a potent LPL inhibitor regulated by glucocorticoid receptor (53) and the fatty acids receptor PPARδ (34, 54) (Supplemental Figure 4A). Exercise increased FFAs and corticosterone concentrations in the circulation (Supplemental Figure 1D and Supplemental Figure 4B). Consistently, exercise significantly increased the transcription of *Angptl4* in WAT (Figure 6A) and in the liver (Supplemental Figure 4C), consistent with previous reports (55, 56). Previous studies reported that liver-derived ANGPTL4 has no effect for peripheral tissue LPL activity, while adipose tissue ANGPTL4 inhibits LPL activity locally (28, 31, 57). We sought to understand the role of adipose tissue ANGPTL4 in response to exercise.

Adipose tissue–specific *Angptl4*-knockout mice (Ad-*Angptl4*^–/–^) had similar ANGPTL3 tissue binding compared with their littermate controls (Supplemental Figure 4, D and E). Like that in wild-type mice, exercise increased the LPL activity of the heart and soleus muscle of Ad-*Angptl4*^–/–^ mice (Figure 6B). In sharp contrast to wild-type mice, exercise increased LPL activity in the WAT of Ad-*Angptl4*^–/–^ mice (Figure 6B). This increased LPL activity was accompanied by decreased ANGPTL3 binding in the tissues (Supplemental Figure 4E). Blocking ANGPTL3 function abolished LPL regulation for the heart and soleus muscle and the WAT of the Ad-*Angptl4*^–/–^ mice in response to exercise (Figure 6, C and D). These data suggest that exercise suppresses WAT LPL activity through coordinated regulation of both hepatic ANGPTL3 and ANGPTL8 and WAT ANGPTL4, with the later playing a dominant role. On the other hand, exercise increases heart and skeletal muscle LPL activity through suppressing hepatic ANGPTL8, which reduces ANGPTL3 tissue binding and increases tissue LPL activity (Figure 6E). Adipose tissue ANGPTL8 was shown to inhibit ANGPTL4 locally (46, 58). We found that exercise also suppressed *Angptl8* transcription in WAT (Supplemental Figure 4F), which may also contribute to the decreased WAT LPL activity in response to exercise. Interestingly, regular exercise training also decreased the circulation levels of the ANGPTL8/ANGPTL3 complex significantly and improved lipid traits (59), suggesting that the ANGPTL families play important roles in response to different types of exercise.

## Discussion

Metabolic flexibility is crucial for health. Its dysfunction is a major driving force for metabolic syndromes, including insulin resistance, ectopic lipid deposition, and type 2 diabetes (35). Substrate switching between carbohydrates and fat lays the groundwork for metabolic flexibility. Exercise elicits a dramatic neuronal-endocrine response, including suppressed insulin secretion and increased stress hormones secretion (60), which plays critical roles in mobilizing stored TG and glycogen (61, 62). Different tissues, such as heart and skeletal muscle, have distinct requirements for energy substrates compared with WAT, necessitating tightly controlled tissue-specific substrate delivery to meet their physiological needs.

Glucose, FFA, and TG are 3 major metabolic fuels in the circulation. Exercise suppresses insulin secretion and limits the glucose uptake in the adipose tissue (63). On the other hand, exercise enhances glucose uptake in the heart and skeletal muscle mainly through muscle contraction, calcium signaling, and AMPK activation (4, 61). By doing this, exercise shifts circulating glucose to heart and skeletal muscles for energy production. Regarding FFA, exercise enhances FFA uptake in skeletal muscle through the activation of transporter protein, such as CD36, at least partially mediated by the calcium signaling and AMPK activation (4). To our knowledge, whether exercise suppresses FFA uptake in WAT remains unclear. However, circulating FFA serves as an important resource for hepatic very-low-density lipoprotein production (64), which is subjected to tissue-specific uptake, as described below.

Circulating TG is carried in hepatic-derived very-low-density lipoprotein and intestinal-derived chylomicrons. It is an important fuel source for peripheral tissues. However, whether and how exercise regulates tissue-specific LPL activity and TG uptake remain obscure (16–19). Most of these studies did not consider the feeding status. The current study found that exercise coordinates with feeding and increases LPL activity and TG uptake in the heart and skeletal muscle by reducing blood insulin levels, which, by cross-talking with the liver, decreases hepatic *Angptl8* transcription and ANGPTL3 tissue binding, resulting in increased tissue LPL activity (Figure 6E). Meanwhile, exercise increases *Angptl4* transcription and inhibits LPL activity in WAT locally. By doing this, exercise shifts circulating TG toward heart and skeletal muscle for energy production in the postprandial state (Figure 6E).

ANGPTL3, ANGPTL4, and ANGPTL8 all contain a conserved LPL binding motif (20). However, how they coordinate with each other and regulate tissue-specific LPL activity remains incompletely understood. Our current study provides compelling evidence that the ANGPTL8/ANGPTL3 complex determines LPL activity in the heart and skeletal muscle, while ANGPTL4 counteracts ANGPTL8/ANGPTL3 function and determines LPL activity in WAT (Figure 6E). This regulatory mechanism may also play important roles in other physiological conditions, such as fasting and cold exposure. Notably, a previous study also reported that ANGPTL4 diverts circulating TG from the resting muscle to the exercising muscle (34), suggesting that there are multilayer regulations for the tissue-specific TG delivery.

ANGPTL3 alone is a poor LPL inhibitor. ANGPTL8 cannot inhibit LPL by itself and functions as a cofactor for ANGPTL3 (20, 43, 45–47). Previous study suggested that ANGPTL8 and ANGPTL3 form a tetramer with 1:3 ratio, which creates a new epitope for LPL inhibition (47). To our knowledge, we show for the first time that ANGPTL8 also functions by recruiting circulation ANGPTL3 to the target tissues for efficient LPL inhibition. Previous study reported that ANGPTL8 promotes the binding of ANGPTL3 to LPL in cultured cells (43). However, a recent study reported that feeding decreases LPL levels in the heart through the ANGPTL8/ANGPTL3 complex (42). Whether ANGPTL8 promotes ANGPTL3 binding in tissues through LPL remains to be investigated. Interestingly, ANGPTL3’s N-terminal binds with tissues completely through ANGPTL8, while its C-terminal binds with tissues independently of ANGPTL8 (Figure 2). This ANGPTL8-independent C-terminal binding provides a plausible explanation for the observation that full-length ANGPTL3 binds with tissues through ANGPTL8 partially (Figure 2). However, this ANGPTL8-independent ANGPTL3 binding does not respond to exercise, suggesting that this part of ANGPTL3 is not required for the exercise-responsive LPL regulation. The physiological functions of these parts of ANGPTL3 and their binding partners in tissues remain unknown.

Insulin has been well known for regulating tissue glucose uptake. Our current study found that insulin also plays critical roles in regulating tissue-specific TG uptake, mainly through the hepatic PI3K/mTOR/CEBPα signaling pathway. CEBPα is a master transcription factor in regulation of hepatic lipid metabolism and responds to hormonal and metabolic regulation (52, 65). Our current study suggested that CEBPα functions downstream of mTOR for ANGPTL8 regulation. However, how mTOR regulates CEBPα expression and activity remain to be investigated. Previous studies also suggest that ANGPTL8 is regulated by CEBPβ and ZNF638 (50, 66). How CEBPα, CEBPβ, and ZNF638 coordinate with each other in response to different nutritional and hormonal signals will need further investigation.

Ectopic lipid deposition, where lipids accumulate in nonadipose tissues, is one of the major reasons for insulin resistance. The role of insulin in regulating tissue-specific TG uptake and substrate switching suggests that insulin resistance will further promote ectopic lipid deposition. This feed-forward loop provides a molecular explanation for the strong association of insulin resistance and ectopic lipid deposition (67). Whether manipulating this pathway will disrupt the feed-forward loop and improve insulin sensitivity remains to be determined.

The role of exercise in preventing and treating a multitude of diseases has garnered increasing attention. Yet, a significant challenge persists — the low adherence to exercise regimens as a form of “exercise medicine.” Thus, what are the molecular mechanisms for exercise benefits, and how to maximize these benefits with similar exercise intensity, have immense interest both within the scientific community and to the general public. Recent studies suggest that exercising at different times of the day has difference effects in improving metabolic health, with postprandial exercise (68–70) having a better outcome than exercise at fasting (71). However, the precise mechanisms remain elusive. Our study, while unraveling pathways and mechanisms governing TG utilization during exercise, also yielded a surprising revelation — exercise fails to increase heart and skeleton muscle TG uptake and utilization during fasting, coinciding with low insulin level. This insulin-dependent regulatory mechanism potentially explains the superior benefits of postprandial exercise.

## Methods

### Sex as a biological variable.

Sex was not considered as a biological variable, and male and female individuals and animals were used in current study. Sex of study participants and mice are provided in figure legends.

### Materials.

We obtained insulin from Novo Nordisk (11061-68-0), Intralipid (20%) from BBCA Pharmaceutical of China, 9,10-[^3^H]triolein from PerkinElmer (NET431005MC), fatty acids free bovine serum albumin (BSA) from Sangon Biotech (A602448), Rapamycin from Selleck (AY-22989), an insulin ELISA kit from EZassay (MS100), a Glucocorticoid ELISA kit from Enzo life (ADI-900-097), heparin from Beijing Wokai Biotechnology (A47476), GW501516 from MCE (HY-10838), and Streptozotocin from MilliporeSigma (S0130). The ANGPTL3 inhibitory antibody has been described previously (32).

### Animals.

Male Sprague-Dawley rats and C57BL/6J wild-type mice were purchased from the Central Disease Control of Hubei province, China. *Angptl3^–/–^* mice were generated by CRISPR/Cas9 technology with 2 gRNA flanking exon 1 and exon 6 on C57BL/6J background by GemPharmatech (Supplemental Figure 4G). F0 mice were crossed with wild-type C57BL/6J mice, and F1 mice were used for further analysis. *Angptl4^fl/+^* mice were obtained from GemPharmatech (strain T016209). The adipocyte-specific *Angptl4*-knockout mice (Ad-*Angptl4^–/–^*) were generated by crossing the *Angptl4^fl/fl^* mice with *Adiponectin*-*Cre* (72). *Angptl8^–/–^* mice (NM-KO-190241) were obtained from Shanghai Model Organisms. SpCas9-transgenic mice were obtained from The Jackson Laboratory (strain 028551). The littermate control mice were used for all studies unless otherwise indicated. Mice and rats were housed in a specific pathogen–free animal facility with a controlled environment (12-h-light/12-h-dark daily cycle, 23°C ± 1°C, 60–70% humidity) at Wuhan University. All animals were fed with standard chow diet unless otherwise indicated.

Before exercise, mouse food intake was synchronized, with 3 days of daytime feeding (9:00 am to 15:00 pm) and night time fasting (15:00 pm to 9:00 am). On each day, 3 hours after food intake (12:00 pm), mice were acclimated to the treadmill by being put in their respective lanes while the belt was still and shock grids were on for 10 minutes. Then, the mice were forced to run for 5 minutes at 10 m/min. On day 4, a single bout aerobic exercise was performed at a postprandial period or at fasting. For postprandial exercise, mice were refed at 9:00 am, and food was removed at 12:00 pm. Half of the mice was subjected to exercise and the other half was left sedentary. All samples were collected at the same time at the end of exercise. For fasting exercise, mice were kept at fasting. Half of mice were subjected to exercise at 12:00 pm, and the other half of mice were left sedentary. All samples were collected at the same time at the end of exercise (Supplemental Figure 1). A single bout aerobic exercise was set to 14 m/min for 3 hours unless otherwise indicated. 14 m/min is about half of the maximum speed for mice used in the study. In some experiments, mice were first infused with adeno-associated virus expressing target cDNA or gRNA through tail vein infusion. Fourteen days later, mice were subjected to food synchronization and exercise as described above.

Mouse energy expenditure was measured with metabolic cages equipped with treadmills (CLAMS, Columbus Instruments). First, mouse food intake was synchronized as described previously. On each day, 3 hours after food intake (12:00 pm), mice were acclimated to the treadmill in metabolic cages by being put in their respective lanes while the belt was still and shock grids were on for 10 minutes. Then, the mice were forced to run for 5 minutes at 10 m/min. On day 4, a single bout of aerobic exercise (14 m/min) was performed at 3 hours after refeeding (postprandial) or at fasting. Respiratory exchange ratio and heat production were calculated according to the instruction.

For exercise with insulin supplementation, mice were synchronized as described above. Three hours after food intake, mice were subjected for exercise at 14 m/min for 1.5 hours. Then, mice were anesthetized with isoflurane, and insulin (1.5 U/Kg) was injected i.p. Mice were forced to run at 14 m/min for another 1.5 hours. Type 1 diabetic mice were generated by a single i.p. injection of streptozotocin (150 mg/kg). The control group was injected with control citrate buffer (pH 4.5). Six days later, mouse food intake was synchronized, and mice were subjected to exercise postprandially as described above.

### Exercise in humans.

Fifteen healthy volunteers were recruited from graduate students at Wuhan University (age, 25 ± 5 years, 8 male individuals and 6 female individuals). On day 1, all individuals ate breakfast at 8:30 am. Then, they rested, and vein blood was draw at 10:00 am. On day 2, all individuals ate similar breakfast at 8:30 am and start running at 9:00 am for 1 hour. Each person ran with moderate intensity at their own comfortable speed (average speed was about 10 km per hour). Vein blood was drawn at the end of exercise.

### Tissue LPL activity.

Tissue LPL activity was measured exactly as described before (26). Briefly speaking, mice were anesthetized with isoflurane. Fresh tissues were collected and minced in cold DMEM with fatty acid–free bovine serum albumin (2%) and heparin (2 U/mL). The tissues were incubated at 37°C for 30 minutes with slow rotation. After spinning down at 6,000*g* for 5 minutes, the medium was collected, snapped frozen with liquid nitrogen, and saved at –80°C. TG lipase activity was measured using a glycerol-stabilized emulsion composed of 9,10-[^3^H]triolein, triolein, and phosphatidylcholine. LPL activity is expressed as the rate of FFAs release (27).

### TG-^3^H-palmitate tissue uptake.

The TG-^3^H-palmitate tissue uptake experiments were performed with intralipids containing 9.10-[^3^H]triolein as the TG emulsion substrate. Briefly, 9.10-[^3^H]triolein was dried under nitrogen gas and then suspended by sonication with 5% of intralipids on ice (50 W power, 30 seconds on, 60 seconds off for 3 cycles). The suspension was diluted 10-fold with saline and was used for tissue uptake assay. For each 20 g of body weight, 200 μL was infused through tail vein (containing 1 mg TG and 2 μCi 9.10-[^3^H]triolein). Ten minutes later, mice were anesthetized with isoflurane and perfused with 10 mL cold saline through the left ventricle unless otherwise indicated. Tissue lipids were extracted with Folch solution and were subjected to scintillation counting. Blood was collected at the end of experiment, and plasma ^3^H activity was expressed as a percentage of the initial injected value. Total blood volume was calculated as 5% of body weight. The initial injected value was expressed as total injected amount divided by the total blood volume.

### [^14^C]Deoxyglucose uptake.

Adeno-associated viruses (AAVs) expressing GFP or human ANGPTL8 (hAN8) with hepatic-specific thyroxine binding globulin (TBG) promoter were infused through the tail vein in *Angptl8^–/–^* mice (1 × 10^12^ particles/mouse). Fourteen days later, mice were subjected to a single bout of exercise (14 m/min, 3 hours) postprandially. The 2-Deoxy-D-glucose uptake was performed during the last hour of exercise as described previously (26). Briefly, for each 20 g body weight, 200 μL saline containing 3 μCi of 2-[1-^14^C]deoxy-D-glucose (PerkinElmer) was injected via the tail vein. Sixty minutes after injection, tissues were collected after extensively perfusion with cold PBS through the left ventricle. Indicated tissues were homogenized, and tissue lysate was subjected to scintillation counting.

### Echocardiography.

AAVs expressing GFP or hAN8 with hepatic-specific TBG promoter were infused through the tail vein in *Angptl8^–/–^* mice (1 × 10^12^ particles/mouse). Fourteen days later, mice were subjected to an exercise challenge for 2 weeks (14 m/min, 2 hours daily postprandially). Transthoracic echocardiography was performed using a VisualSonics Vevo 3100 system equipped with MX550D transducer (FUJIFILM Visual Sonics). Body temperature was under controlled conditions during echocardiogram acquisition. Systolic function was obtained from short-axis M-mode at the midventricular level, as indicated by the presence of papillary muscles. Anesthesia was induced by 2% isoflurane mixed with 1 L/min air and confirmed by lack of response to firm pressure on one of the hind paws. Isoflurane was reduced to 0.5%–1% mixed with 1 L/min air to maintain a heart rate in the range of 550–610 beats per minute during imaging collection. Diastolic function was measured through apical 4-chamber views using pulsed-wave and tissue Doppler imaging at the level of the mitral valve. Anesthesia was induced by 4% isoflurane mixed with 1 L/min air and confirmed by lack of response to firm pressure on one of the hind paws. Isoflurane was reduced to 1.5%–2% to maintain a heart rate in the range of 340–400 beats per minute during imaging collection. At the end of the procedures, all mice were recovered from anesthesia without difficulties. Parameters collected include heart rate, left ventricular end-diastolic diameter, end-diastolic interventricular septal wall thickness, left ventricular end-diastolic posterior wall, peak Doppler blood inflow velocity across the mitral valve during early diastole (E), peak Doppler blood inflow velocity across the mitral valve during late diastole (A), peak tissue Doppler of myocardial relaxation velocity at the mitral valve annulus during early diastole (E′), and peak tissue Doppler of myocardial relaxation velocity at the mitral valve annulus during late diastole (A′). Left ventricular fractional shortening, left ventricular ejection fraction, and the E/E′ were analyzed using Vevo Analysis software according to the manufacturer’s instructions (FUJIFILM Visual Sonics). All parameters were measured at least 3 times, and means are presented.

### Blood chemistry.

Blood was collected and clotted at room temperature for 60 minutes. Serum was isolated by centrifugation at 3,500*g* for 10 minutes at 4°C. TG and cholesterol were measured using an enzymatic kit from Shanghai Kehua Bio-Engineering Co. Nonesterified fatty acids were measured using the HR Series NEFA-HR Kit (Wako). Lactate was detected using colorimetric kits (Elabscience, E-BC-K044-M). Blood glucose was measured using a glucometer (Contour; Bayer) by eye bleeding.

### Cell culture and virus production.

293T cells were cultured in high glucose DMEM (Life Technologies, 12800-082) with 10% FBS (ExCell Bio Inc., FSP500), 100 units/mL sodium penicillin (Sangon Biotech, A600135), and 100 μg/mL streptomycin sulfate (Sangon Biotech, A610494) at 37°C with 5% CO_2_. EDTA-free protein inhibitor cocktail was purchased from Selleck (B14001). Polyethylenimine was obtained from Polysciences (24765-2).

Rat primary hepatocytes were isolated from rats ad libitum by the collagenase method and cultured as described previously (73). Three hours after plating, cells were washed twice with PBS, after which M199 medium was added to cells. (Gibco, 31100019) supplemented with 100 nM dexamethasone (MilliporeSigma, D4902), 100 nM 3,3′,5-Triiodo-L-thyronine (MilliporeSigma, T2877), 100 units/mL sodium penicillin (Sangon Biotech, A600135) and 100 μg/mL streptomycin sulfate (Sangon Biotech, A610494). Sixteen hours later, cells were treated with wortmannin (200 nM) or rapamycin (100 nM) for 30 minutes and followed by insulin treatment (100 nM) for another 3 hours. Then, cells were harvested for further analysis. In some experiments, rat primary hepatocytes were transfected with double-stranded siRNA oligos with Lipofectamine RNAiMAX (Invitrogen, 13778075) according to the manufacturer’s instruction. Then, cells were incubated for 48 hours before insulin treatment. siRNA oligos were synthesized by Genepharma, and the sequences are listed in Supplemental Table 1.

Mouse primary hepatocytes were isolated from refed mice by the collagenase method and cultured exactly as described previously (74). Three hours after plating, cells were washed twice with PBS and changed to high glucose DMEM (Life Technologies, 12800-082) with 10% FBS (ExCell Bio Inc., FSP500), 100 units/mL penicillin, and 100 μg/mL streptomycin sulfate (Gibco, 15140-122) with the dexamethasone or GW501516 for 3 hours. Then, cells were harvested for further analysis.

AAVs were produced in 293T cells as described previously (72). Expression vector with TBG promoter was used for hepatic-specific expression of human ANGPTL8 in mice. CRISPR/Cas9-mediated *Cebpα* knockdown was performed in SpCas9-transgenic mice. AAVs expressing gRNA targeting mouse *Cebpα* was expressed with U6 promoter. gRNA sequence is listed in Supplemental Table 1.

### Immunoblot.

Immunoblot was performed as previously described (74), with the exception that the cells and tissues were lysed in lysis buffer (1% Triton, 50 mM tris-HCL, 150 mM NaCl, 5 mM EDTA, 5 mM EGTA). All tissues were collected after extensive perfusion with ice-cold saline through the left ventricle. Serum was diluted 20-fold with saline before sample buffer was added. Nonreducing sample buffer was used for all samples except for immune blot of Akt and S6K together with their respect controls. To detect human ANGPTL8, liver lysate and serum were immunoprecipitated with anti-Flag M2 beads (MilliporeSigma). Then, the eluted samples were subjected to immunoblot analysis with anti-ANGPTL8 antibody as described previously (20).

The following antibodies were used in current study: Anti-angptl3 antibody (R&D Systems, AF136), Anti-Calnexin antibody (Proteintech, 10427-2-AP), Anti-Vcl antibody (ABclonal, A2752), Anti-Flag antibody (Medical & Biological Laboratories, PM020), Anti-AKT antibody (Cell Signaling Technology, 88800), Anti p-AKT antibody (Cell Signaling Technology, 4060), Anti-S6K antibody (Cell Signaling Technology, 2708), Anti–p-S6K antibody (Cell Signaling Technology, 97596), and Anti-cebpα antibody (Cell Signaling Technology, 8178).

### Real-Time PCR.

Total RNA was extracted from tissues or cells with TRI Reagent (MilliporeSigma, T9424). The concentration and quality were measured with NanoDrop (Thermo Scientific). Two micrograms of total RNA was used for cDNA synthesize with a Reverse Transcription Kit (Yeasen Biotech, 11121ES60). Real-time PCR was performed with SYBR Green master mix (Yeasen Biotech, 11201ES08) together with indicated primers for each gene. *36B4* was used as internal control, except when otherwise indicated. The average expression level for the control group was set to 1. All primers are listed in Supplemental Table 1.

### Angptl8 transcription analysis.

Total RNA was extracted from tissues with TRI Reagent (MilliporeSigma, T9424). RNA-Seq was performed as described previously (27). KEGG pathway enrichment analysis was performed with the online tool Dr.Tom (https://biosys.bgi.com), created by the Beijing Genomics Institute, according to their instructions.

Transcription factor binding analysis was performed with the Cistrome DB Toolkit (http://dbtoolkit.cistrome.org/). Transcription factors bound within the 1 Kb and 10 Kb regions of the transcription start site of human *ANGPTL8* and mouse *Angptl8* were selected and were subjected for further functional verification.

### Statistics.

All data are expressed as mean ± SEM, and *P* values were calculated using unpaired 2-tailed Student’s *t* test in GraphPad Prism 8.0.1 unless otherwise indicated. Data are shown as the mean ± SEM. *P* values of less than 0.05 were considered significant.

### Study approval.

Human samples were used with approval from the Biomedical Ethic Committee of Wuhan University (protocol IRB2022014). All animal protocols were approved by the Institutional Animal Use and Care Committee of College of Life Sciences, Wuhan University (protocol WDSKY0201504-2).

### Data availability.

Liver RNA-Seq data are available at GEO (accession GSE269016). Values for all data points in graphs are available in the Supporting Data Values file.

## Author contributions

Conceptualization was contributed by YW, XML, and YLZ. Methodology and investigation were contributed by XML, YLZ, BQH, LL, YL, YFM, SJK, QL, LKK, KH, BLS, YL, YW. Transcriptomic analysis was contributed by XML. Writing of the original draft was contributed by YW, XML and YLZ. Supervision was contributed by YW. Funding acquisition was contributed by YW.

## Supplementary Material

Supplemental data

Unedited blot and gel images

Supporting data values

## Version 1 08/22/2024

Electronic publication
