# Supplementary material for: Postprandial exercise regulates tissue-specific triglyceride uptake through angiopoietin-like proteins
Source: JCI Insight. 2024 Aug 22;9(16):e181553. doi: 10.1172/jci.insight.181553 (PMC11343597; doi:10.1172/jci.insight.181553)
Supplement: Supplemental data [file jciinsight-9-181553-s159.pdf]

Supplementary Materials for

Postprandial exercise regulates tissue-specific triglyceride uptake through Angiopoietin-like proteins

Xiaomin Liu[#1], Yiliang Zhang[#1], Bingqian Han[1], Lin Li[1], Ying Li[1], Yifan Ma[1], Shijia Kang[1], Quan Li[1], Lingkai Kong[1], Kun Huang[2], Bao-liang Song[1], Yong Liu[1], Yan Wang*[1]

Corresponding author: Wang.y@whu.edu.cn

The PDF file includes:

Supplementary Figure 1-4

Supplementary Table 1

Supplementary Figure1

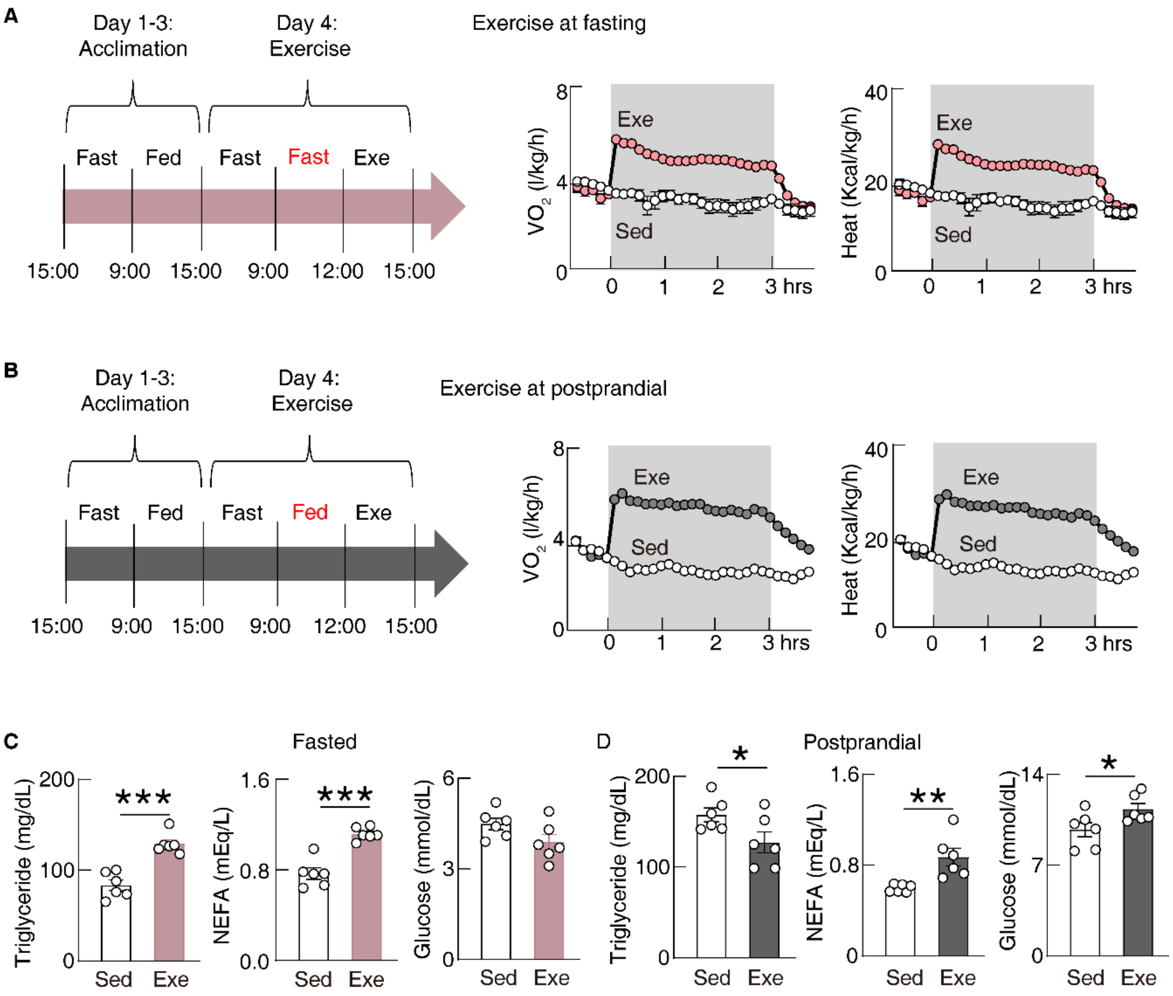

Supplementary Figure1. Metabolic characterization of mice exercised postprandially or at fasting. (**A** and **B**) Exercise protocol, VO_2 and heat production in mice used in Figure 1A and 1B respectively. Mice were first synchronized with food intake and acclimated on treadmill for 3 days. On day4, a single bout of aerobic exercise (14 m/min) was performed with treadmills in metabolic cage at fasting or at postprandial condition as described in *Methods*. (**C** and **D**) Blood chemistry of mice exercised at fasting or at postprandial as in panel A and B respectively (n=6 males/group, 8-10 weeks). All experiments were repeated with similar results. Data are expressed as means ± SEM. *$p < 0.05$, ** $p < 0.01$, *** $p < 0.001$. All abbreviations are the same as in Figure 1.

Supplementary Figure2

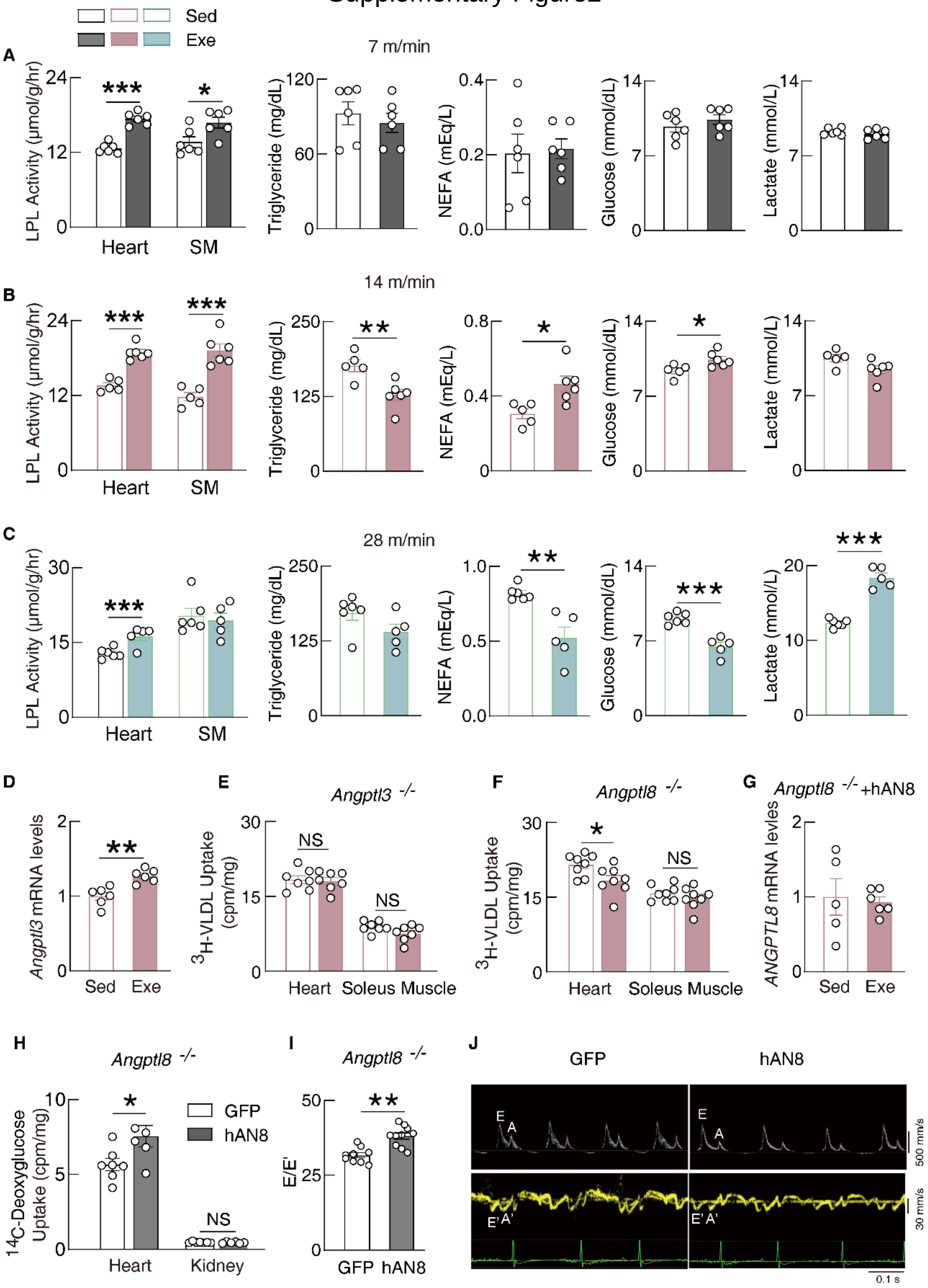

Supplementary Figure2. (**A-C**) Tissue LPL activity and blood chemistry of mice exercised with indicated speed for 3 hours postprandially (n=6 males/group, 8-10W. SM: Soleus muscle). (**D**) Hepatic *Angptl3* mRNA level in mice used in Figure1C. (**E**) Tissue triolein-[^{3}H]palmitate uptake in *Angptl3*$^{-/-}$

mice following postprandial exercise (n=7 males/group, 8-9 weeks). (**F**) Tissue triolein-[^{3}H]palmitate uptake in *Angptl8*$^{-/-}$ mice following postprandial exercise (n=8 males/group, 17-20 weeks). (**G**) Human *ANGPTL8* mRNA level in liver of mice used in Figure 2**G**. (**H**) Tissue ^{14}C-Deoxyglucose uptake in *Angptl8*$^{-/-}$ mice expressing GPF or human ANGPTL8 (hAN8) and exercised postprandially (n=6-7 females/group, 8-10 weeks). (**I**) Heart diastolic function in mice used in Figure 2**I**. (E: peak Doppler blood inflow velocity across the mitral valve during early diastole, E': peak tissue Doppler of myocardial relaxation velocity at the mitral valve annulus during early diastole). (**J**) Representative imaging of pulsed-wave Doppler (top) and tissue Doppler (bottom) tracings of panel **I** Data are expressed as means ± SEM. * $p < 0.05$, ** $p < 0.01$, *** $p < 0.001$. All experiments were repeated with similar results. All abbreviations are the same as in Figure 1.

Supplementary Figure3

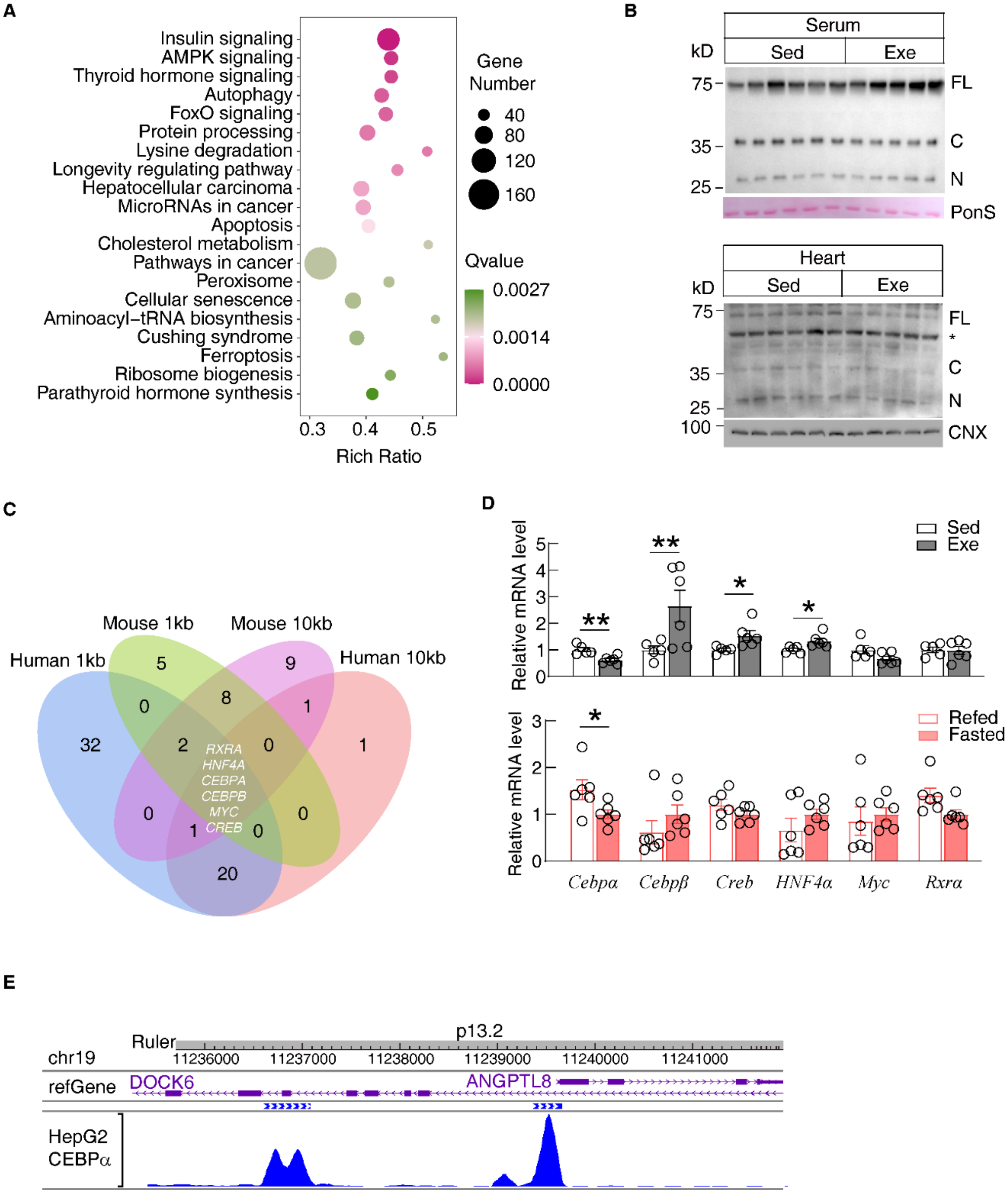

Supplementary Figure3. (**A**) KEGG pathway analysis for hepatic genes in mice following postprandial exercise. Liver samples from mice used in Figure 1C were subjected to RNA sequencing and pathway analysis was performed as described in *Methods*. (**B**) ANGPTL3 protein levels in serum and heart of mice that subjected for exercise at fasting state. The fasting exercise was conducted the same as in Figure 1A (N=5-6 Male/group, 10 weeks, All abbreviations are the same as in Figure 1). *Non-specific band. (**C**) Transcription factor binding motif analysis for human *ANGPTL8* and mouse *Angptl8*. ChIP-seq data were obtained from Cistrome DB and analyzed as described in *Methods*. Transcription factor

binding motif near the start code of *ANGPTL8* and *angptl8* were overlaid together. (**D**) Hepatic gene expression in mice following postprandial exercise or subjected to overnight fasting at sedentary (n=6-7 males/group, 8-10W). Exercise was performed exactly the same as in Figure 1B. Hepatic RNA was extracted and subjected for RT-PCR analysis as described in *Methods*. (**E**) ChIP-seq analysis for ANGPTL8 promoter with CEBPα antibody. Data were obtained from Cistrome DB as described in *Methods*. Data are expressed as means ± SEM. **$p < 0.01$, *** $p < 0.001$. Data are expressed as means ± SEM.

Supplementary Figure4

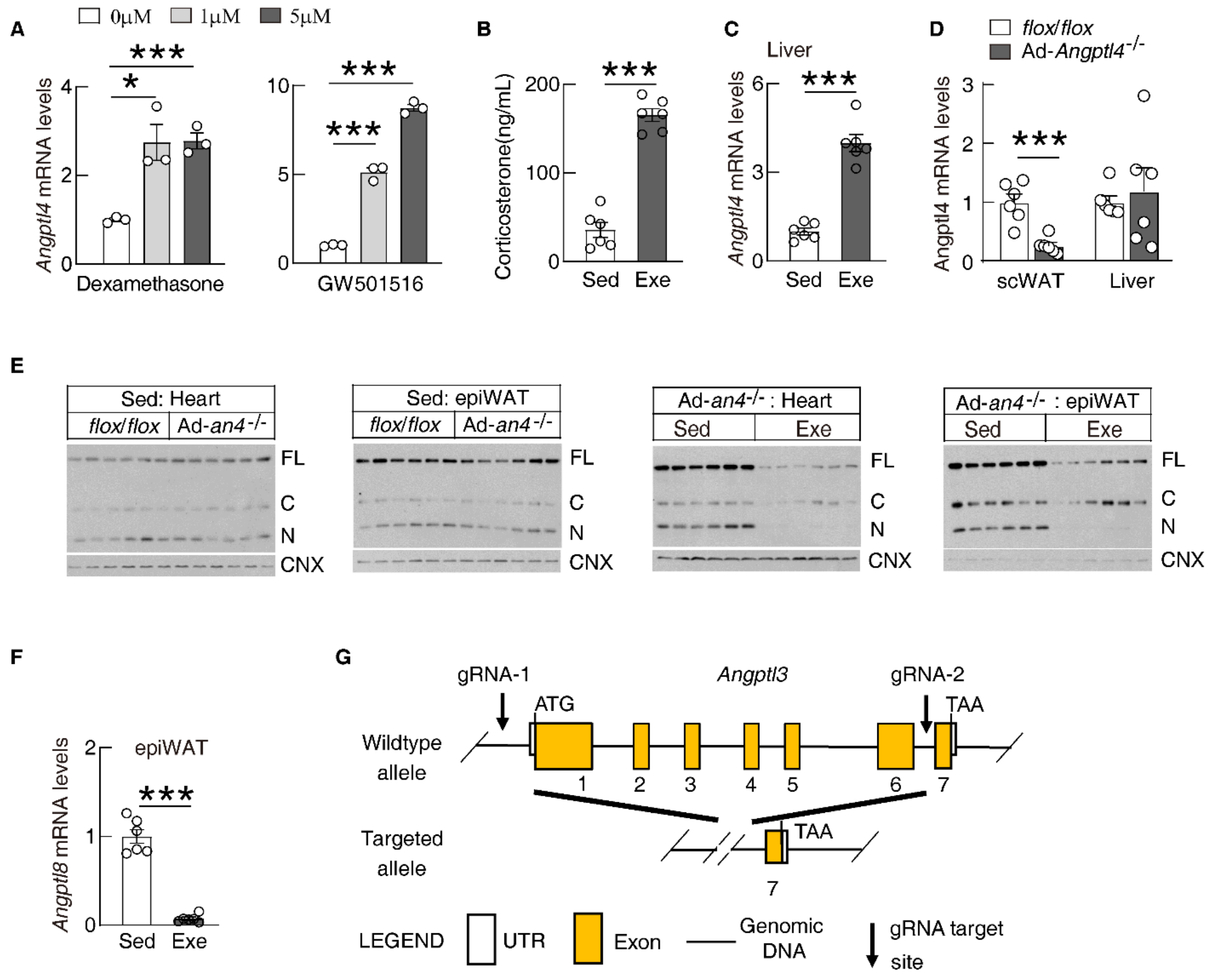

Supplementary Figure4. (**A**) *Angptl4* transcriptional level in primary hepatocytes with indicated treatments. Mouse primary hepatocytes were isolated and treated as described in *Methods*. RNA was extracted and *angptl4* transcriptional level was analyzed with real time PCR. GW501516 is a Pparβ/δ specific agonist. (**B**) Circulation corticosterone levels in mice following postprandial exercise (n=6 males/group, 8-10W). (**C**) Hepatic *Angptl4* transcriptional level in mice following postprandial exercise (n=6 males/group, 8-10W). (**D**) *Angptl4* transcriptional level in adipose tissue-specific *angptl4* knockout mice (Ad-$angptl4^{-/-}$) (n=6 males/group, 7-10W). (**E**) ANGPTL3 protein levels in Ad-$Angptl4^{-/-}$ mice (Ad-$an4^{-/-}$) and littermate control wild-type (WT) mice with or without postprandial exercise (n=6 males/group, 7-10W). (**F**) *Angptl8* transcriptional level in epiWAT of mice following postprandial exercise (n=6 males/group, 7-10W). (**G**), Schematic diagram showing the generation of $angptl3^{-/-}$ mice with CRISPR/Cas9 system as described in *Methods*. (epiWAT: epididymal white adipose tissue, scWAT: subcutaneous white adipose tissue). Data are expressed as means ± SEM. *$p < 0.05$, ***$p < 0.001$. All abbreviations are the same as in Figure 1.

Supplementary table 1: This table contains sequence information used in this study.

**Primers for qPCR**		
**Human**	**Forward**	**Reverse**
36B4	TGCATCAGTACCCCATTCTATCA	AAGGTGTAATCCGTCTCCACAGA
ANGPTL8	GCAAGCCTGTTGGAGACTCAG	CTGTCCCGTAGCACCTTCT
**Mouse**	**Forward**	**Reverse**
36B4	CACTGGTCTAGGACCCGAGAAG	GGTGCCTCTGGAGATTTTCG
Angptl3	AGCAAGACAACAGCATAAGAGAACTC	CTGAGCTGCTTTTCTATTTCTTTTATCTG
Angptl4	GCCTTTCCCTGCCCTTCTC	GATTGGAATGGCTACAGGTACCA
Angptl8	ACATGGCTGTGCTTGCTCTCT	CAAATTCTTGGTGGGCTTGAC
Cebpα	GCGGGAACGCAACAACATC	GTCACTGGTCAACTCCAGCAC
Cebpβ	CGCCTTTAGACCCATGGAAG	CCCGTAGGCCAGGCAGT
RXRα	CAGTACGCAAAGACCTGACCTACA	GTTCCGCTGTCTCTTGTCGAT
Hnf4a	ACTGTCCAGAGCTAGCGGAGAT	GCAGGCATATTCATTGTCATCAA
Creb	GAGCAGACAACCAGCAGAGT	TGGATAACTGATGGCTGGGC
Myc	CGGTTCCTTCTGACAGAACTGA	CCAGCCAAGGTTGTGAGGTT
**Rat**	**Forward**	**Reverse**
36B4	TTCCCACTGGCTGAAAAGGT	CGCAGCCGCAAATGC
Angptl8	AGCCGGCCCAATATGAAGAG	GCTGCACTTGTAGTCTCCGT

Cebpα	GGTTTAGGGTCGCTGGATCTC	GGCGACACCAGAATCTCCTAGT

siRNA sequences for rat *Cebpα*.

Rat	Forward	Reverse
siCebpα-1	CACGAGACGUCUAUAGACATT	UGUCUAUAGACGUCUCGUGTT
siCebpα-2	CGGUGGAUAAGAACAGCAATT	UUGCUGUUCUUAUCCACCGTT

gRNA sequence for mouse *Cebpα*

Target gene	**gRNA sequence**
Cebpα	AGAAGTCGGCCGACTCCATG
