# Supplementary material for: Postprandial exercise regulates tissue-specific triglyceride uptake through angiopoietin-like proteins
Source: JCI Insight. 2024 Aug 22;9(16):e181553. doi: 10.1172/jci.insight.181553 (PMC11343597; doi:10.1172/jci.insight.181553)
Supplement: Unedited blot and gel images [file jciinsight-9-181553-s160.pdf]

Fig 1C Serum

Serum	
Sed	Exe

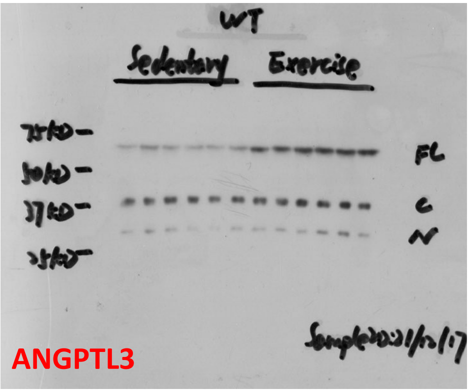

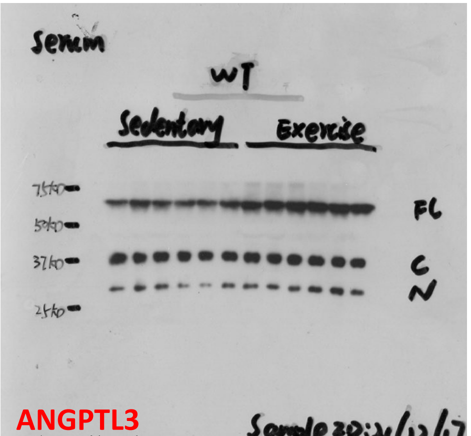

1st antibody:AF136,1:1000

2ed antibody: anti-goat, 1:4000

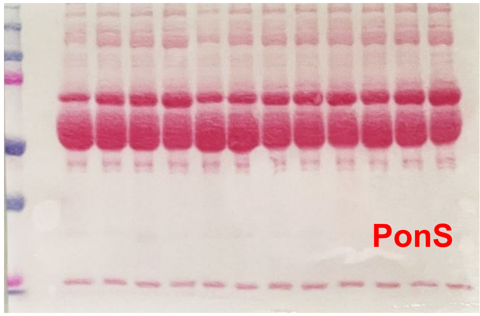

Fig 1C Heart

Heart		K
Sed	Exe	O

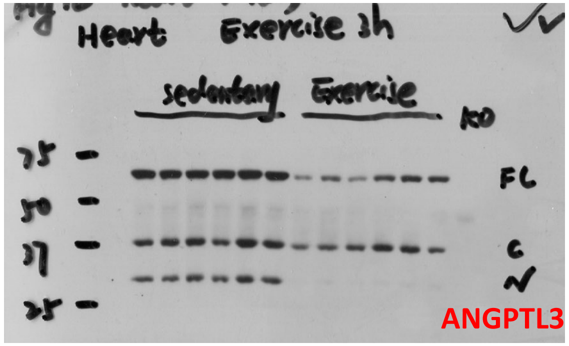

1st antibody:AF136,1:1000

2ed antibody: anti-goat, 1:4000

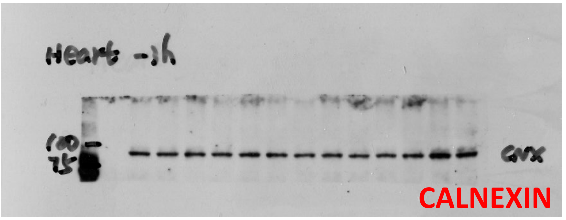

1st antibody:anti-calnexin,1:5000

2ed antibody: anti-rabbit, 1:100000

Fig 1C SM

Solus Muscle		K
Sed	Exe	O

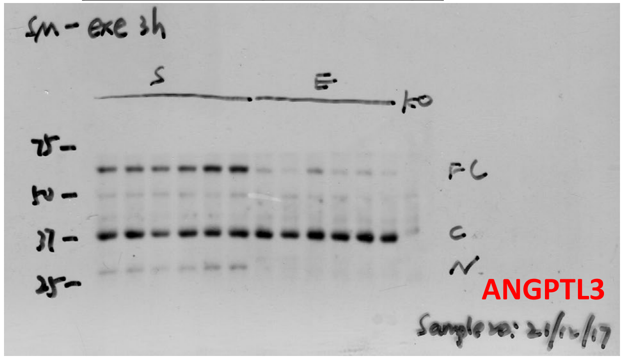

1st antibody:AF136,1:1000

2ed antibody: anti-goat, 1:4000

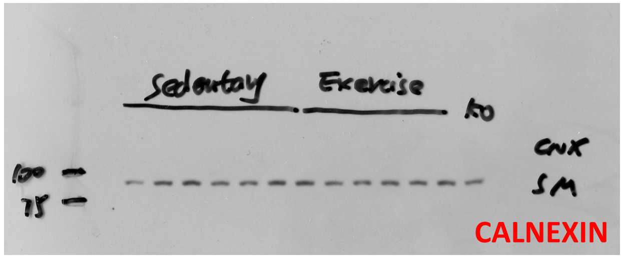

1st antibody:anti-calnexin,1:5000

2ed antibody: anti-rabbit, 1:100000

Fig 2A Serum

Sedentary: Serum	
WT	*Angptl8*$^{-/-}$

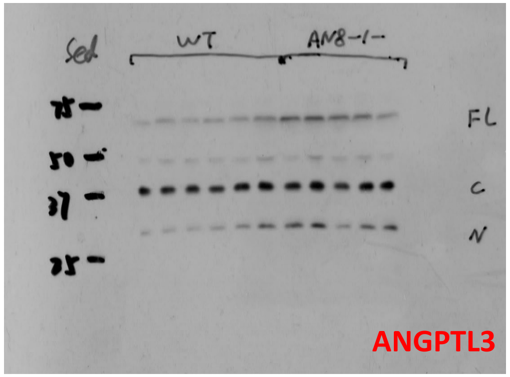

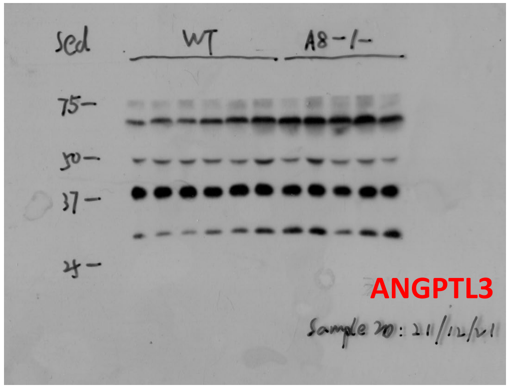

1st antibody:AF136,1:1000
2ed antibody: anti-goat, 1:4000

Sedentary: Serum	
WT	*Angptl8*$^{-/-}$

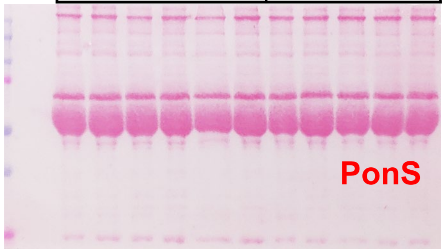

Fig 2A Heart

Sedentary: Heart	
WT	*Angptl 8*$^{-/-}$

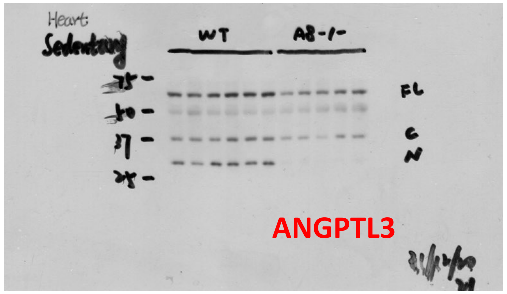

1st antibody:AF136,1:1000
2ed antibody: anti-goat, 1:4000

Sedentary: Heart	
WT	*Angptl8*$^{-/-}$

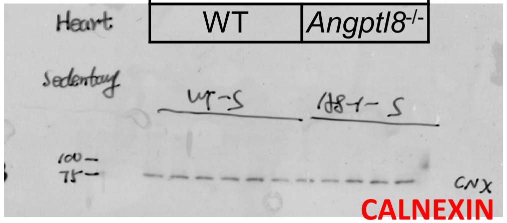

1st antibody:anti-calnexin,1:5000
2ed antibody: anti-rabbit, 1:100000

Fig 2B Serum

WT: Serum	
Sed	Exe

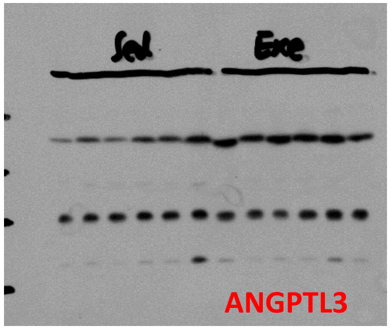

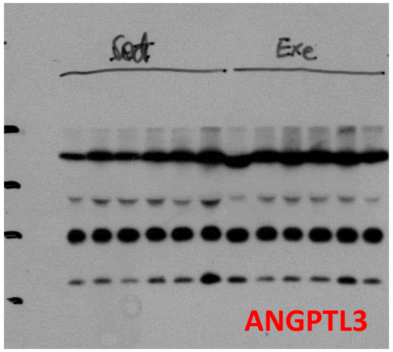

1st antibody:AF136,1:1000
2ed antibody: anti-goat, 1:4000

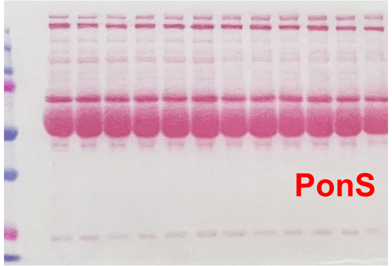

Fig 2B Heart

WT: Heart	
Sed	Exe

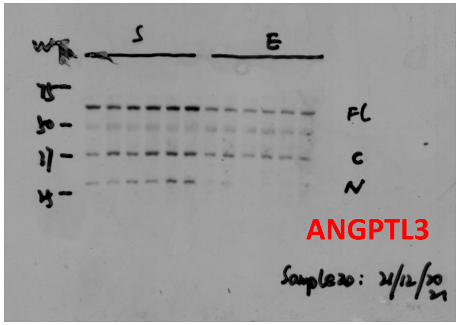

1st antibody:AF136,1:1000
2ed antibody: anti-goat, 1:4000

WT: Heart	
Sed	Exe

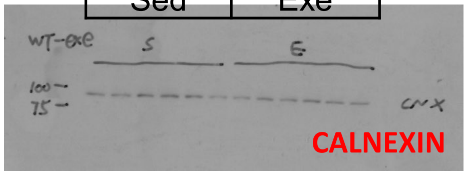

1st antibody:anti-calnexin,1:5000
2ed antibody: anti-rabbit, 1:100000

Fig 2C Serum

Angptl8$^{-/-}$: Serum	
Sed	Exe

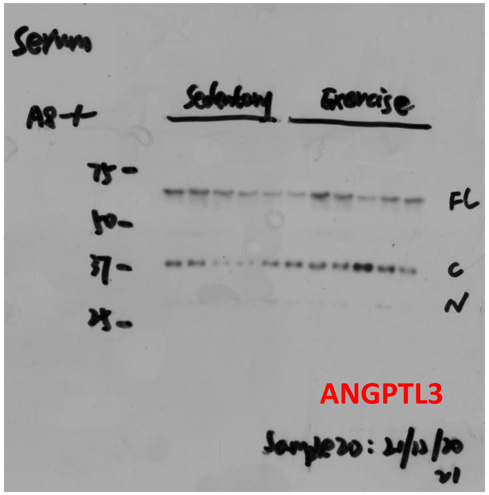

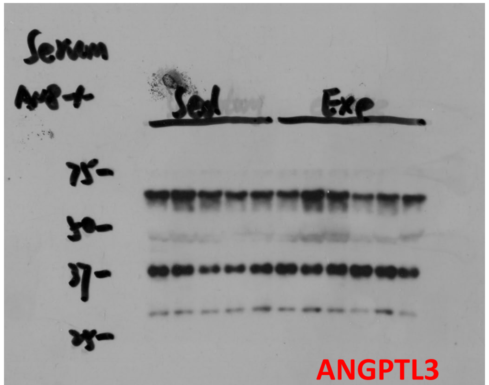

1st antibody:AF136,1:1000
2ed antibody: anti-goat, 1:4000

Angptl8$^{-/-}$: Serum	
Sed	Exe

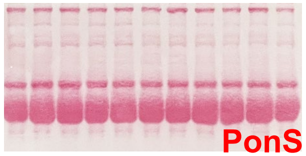

Fig 2C Heart

Angptl8-/-:Heart	
Sedentary	Exercise

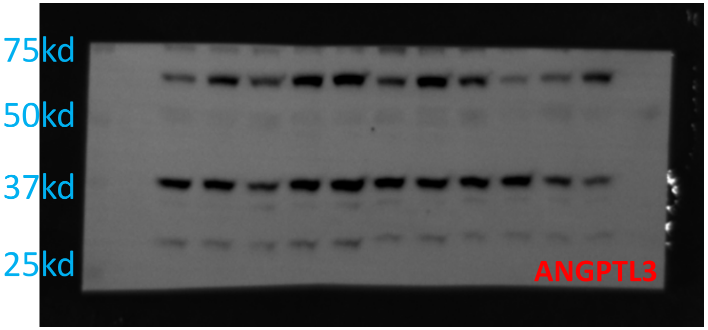

1st antibody:AF136,1:1000
2ed antibody: anti-goat, 1:4000

Angptl8-/-:Heart	
Sedentary	Exercise

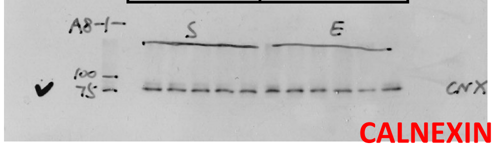

1st antibody:anti-calnexin,1:5000
2ed antibody: anti-rabbit, 1:100000

Fig 2F

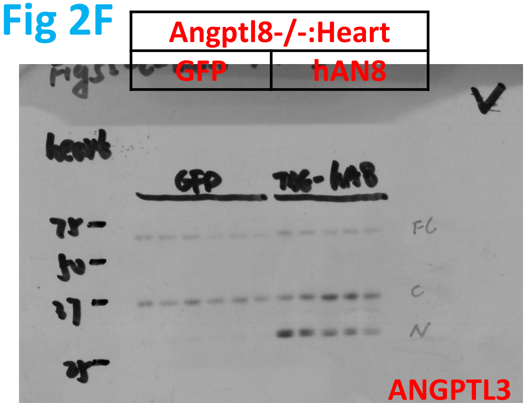

1st antibody:AF136,1:1000
2ed antibody: anti-goat, 1:4000

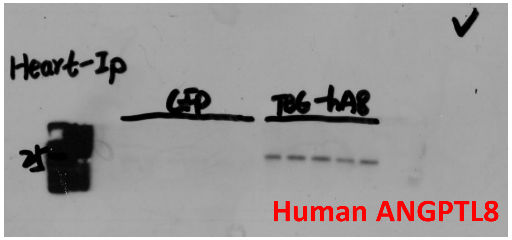

1st antibody:anti-flag,1:1000
2ed antibody: anti-rabbit, 1:100000

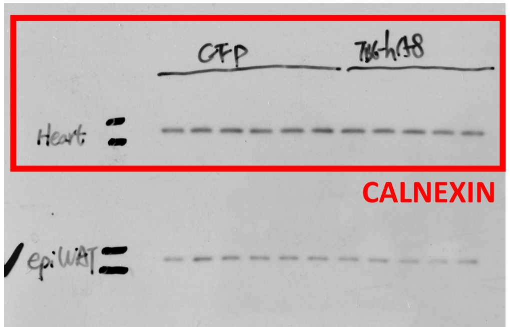

1st antibody:anti-calnexin,1:5000
2ed antibody: anti-rabbit, 1:100000

Fig 2G

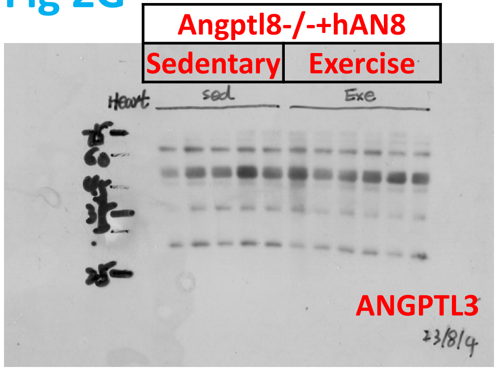

1st antibody:AF136,1:1000
2ed antibody: anti-goat, 1:4000

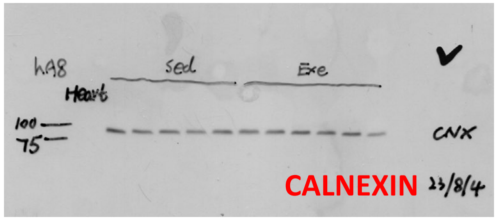

1st antibody:anti-calnexin,1:5000
2ed antibody: anti-rabbit, 1:100000

Fig 3B

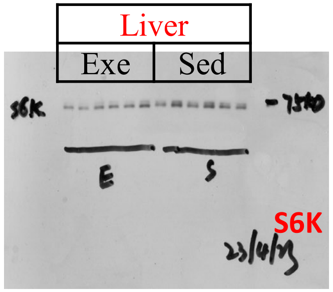

1st antibody:anti-S6K,1:1000
2ed antibody: anti-rabbit, 1:50000

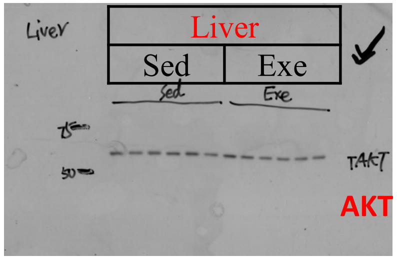

1st antibody:anti-AKT,1:2000
2ed antibody: anti-rabbit, 1:50000

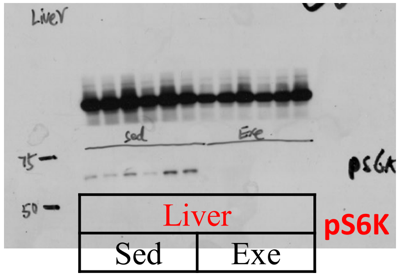

1st antibody:anti-pS6K,1:1000
2ed antibody: anti-rabbit, 1:50000

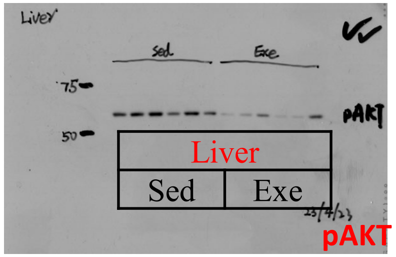

1st antibody:anti-pAKT,1:1000
2ed antibody: anti-rabbit, 1:50000

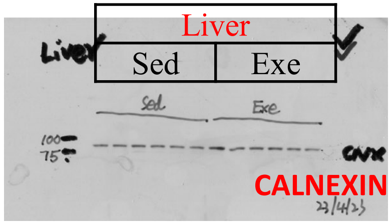

1st antibody:anti-calnexin,1:5000
2ed antibody: anti-rabbit, 1:100000

Fig 3D-Serum

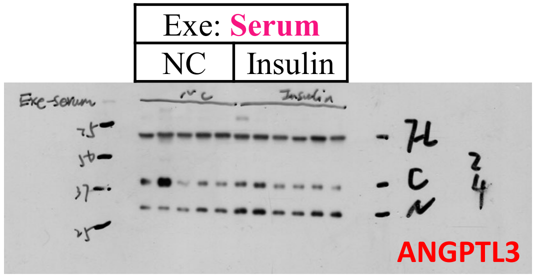

1st antibody:AF136,1:1000
2ed antibody: anti-goat, 1:4000

Fig 3D-Heart

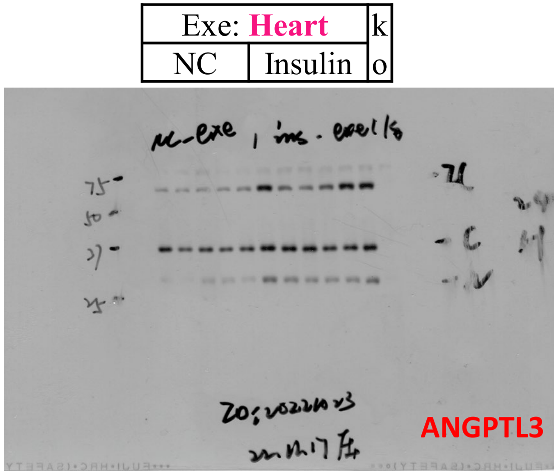

1st antibody:AF136,1:1000
2ed antibody: anti-goat, 1:4000

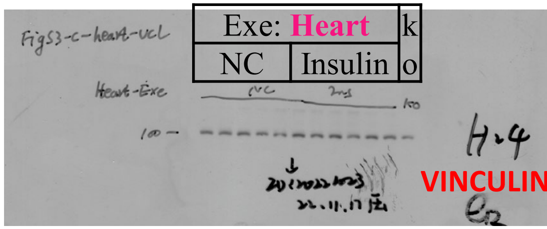

1st antibody:anti-vinculin,1:2000
2ed antibody: anti-rabbit, 1:100000

Fig 4A

Liver	
Sedentary	Exercise

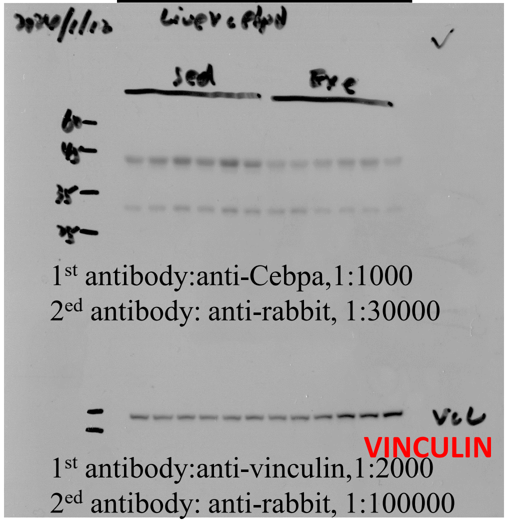

1st antibody:anti-Cebpa,1:1000
2ed antibody: anti-rabbit, 1:30000

1st antibody:anti-vinculin,1:2000
2ed antibody: anti-rabbit, 1:100000

Liver	
Sedentary	Exercise

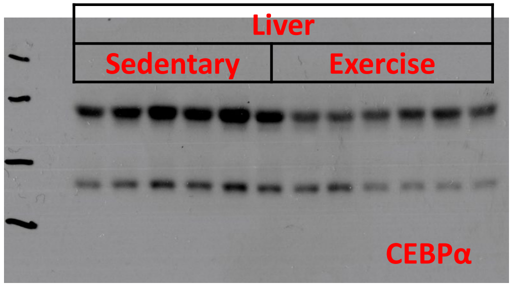

1st antibody:anti-Cebpa,1:1000
2ed antibody: anti-rabbit, 1:30000

Fig 4B

Liver	
NC	sgCebpa

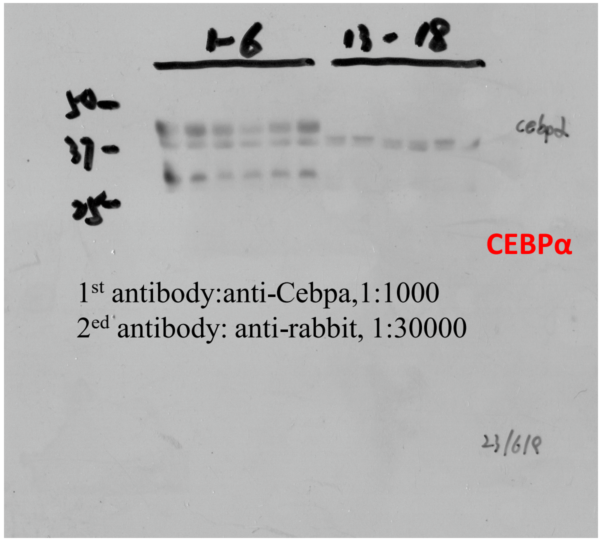

1st antibody:anti-Cebpa,1:1000
2ed antibody: anti-rabbit, 1:30000

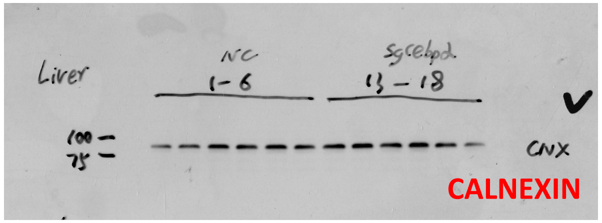

1st antibody:anti-calnexin,1:5000
2ed antibody: anti-rabbit, 1:100000

Fig 4D

Rat primary hepatocyte			
NC	Ra p	Ins	Ra p+I ns

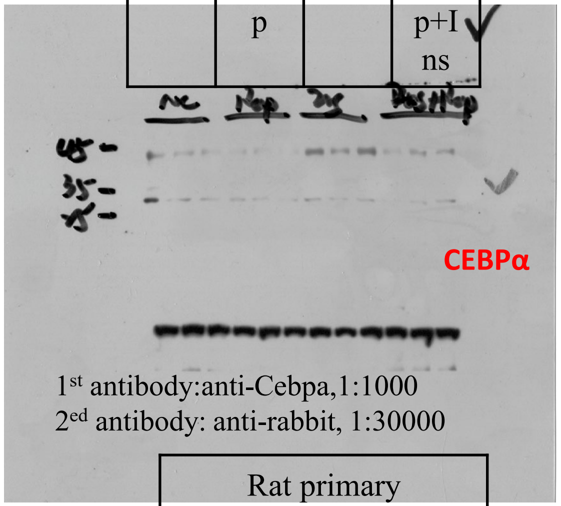

1st antibody:anti-Cebpa,1:1000
2ed antibody: anti-rabbit, 1:30000

Rat primary hepatocyte			
N C	Ra p	Ins	Ra p+ Ins

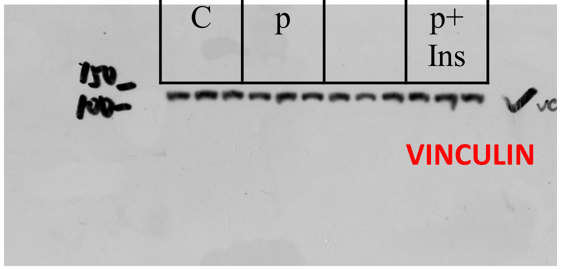

1st antibody:anti-vinculin,1:2000
2ed antibody: anti-rabbit, 1:100000

Fig 5C

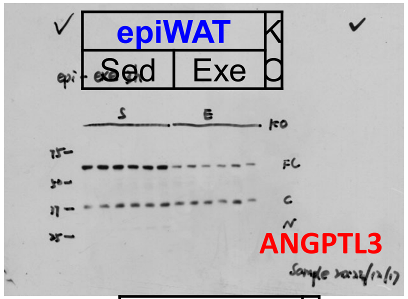

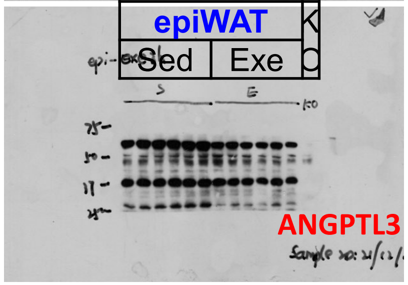

1st antibody:AF136,1:1000
2ed antibody: anti-goat, 1:4000

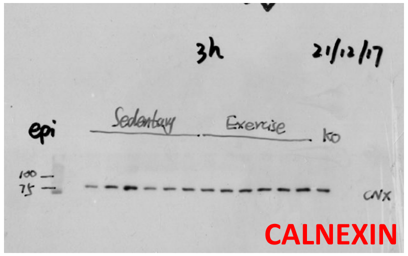

1st antibody:anti-calnexin,1:5000
2ed antibody: anti-rabbit, 1:100000

Fig 5D

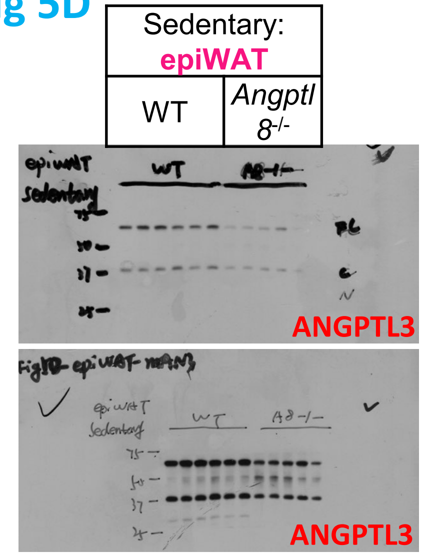

1st antibody:AF136,1:1000
2ed antibody: anti-goat, 1:4000

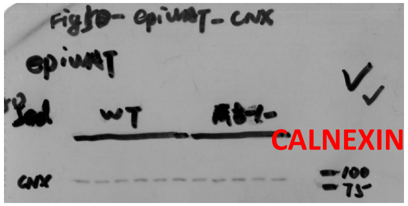

1st antibody:anti-calnexin,1:5000
2ed antibody: anti-rabbit, 1:100000

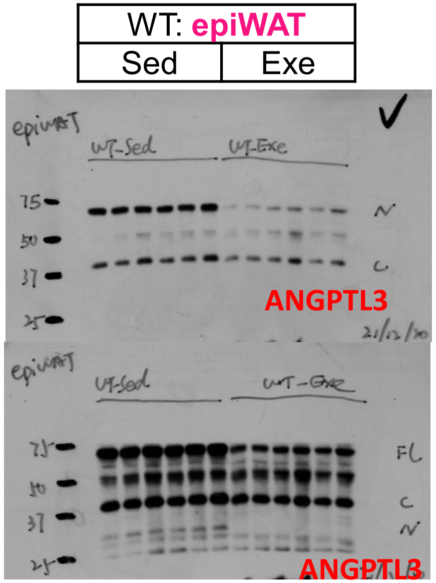

1st antibody:AF136,1:1000
2ed antibody: anti-goat, 1:4000

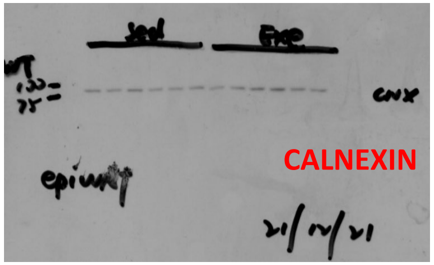

1st antibody:anti-calnexin,1:5000
2ed antibody: anti-rabbit, 1:100000

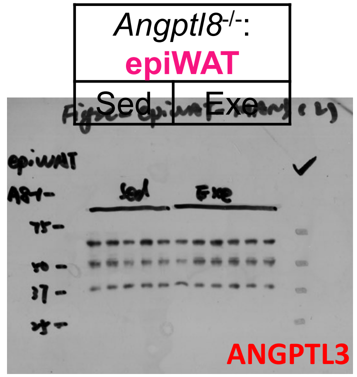

1st antibody:AF136,1:1000
2ed antibody: anti-goat, 1:4000

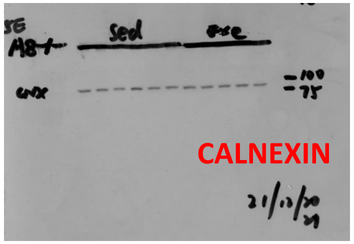

1st antibody:anti-calnexin,1:5000
2ed antibody: anti-rabbit, 1:100000

Fig 5E

Angptl8-/- :epiWAT	
GFP	hAN8

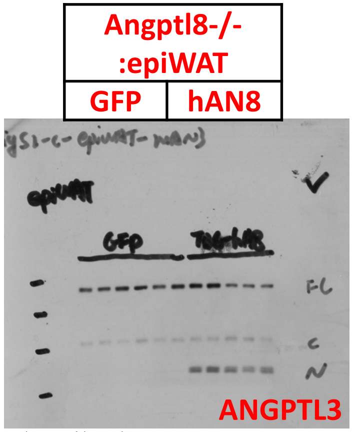

1st antibody:AF136,1:1000
2ed antibody: anti-goat, 1:4000

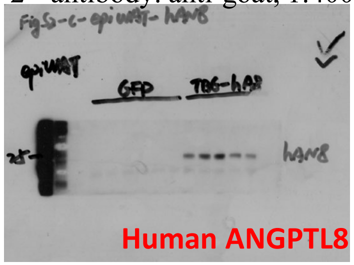

1st antibody:anti-flag,1:1000
2ed antibody: anti-rabbit, 1:100000

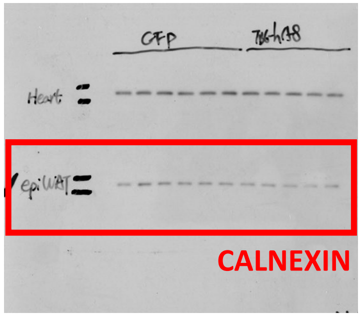

1st antibody:anti-calnexin,1:5000
2ed antibody: anti-rabbit, 1:100000

Fig 5F

Angptl8-/-+hAN8	
Sedentary	Exercise

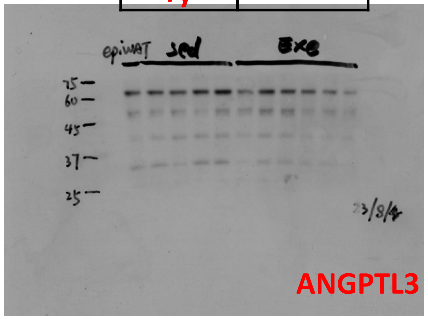

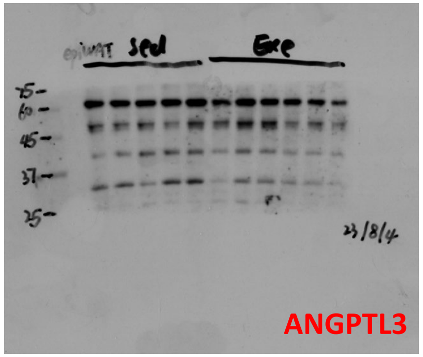

1st antibody:AF136,1:1000
2ed antibody: anti-goat, 1:4000

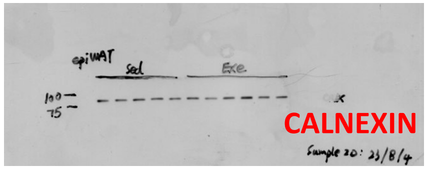

1st antibody:anti-calnexin,1:5000
2ed antibody: anti-rabbit, 1:100000

Fig S3B

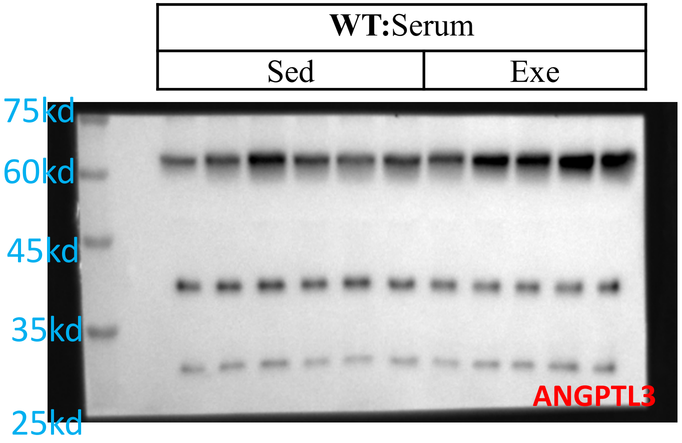

1st antibody:AF136,1:1000
2ed antibody: anti-goat, 1:4000

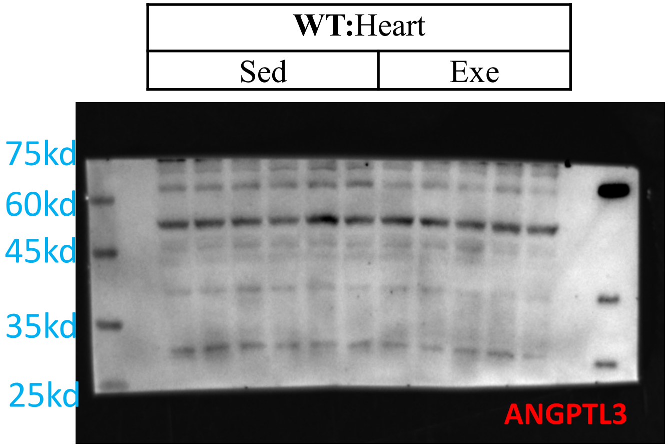

1st antibody:AF136,1:1000
2ed antibody: anti-goat, 1:4000

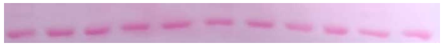

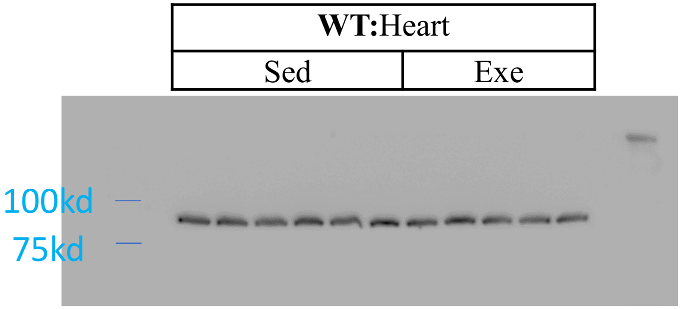

Fig S4E

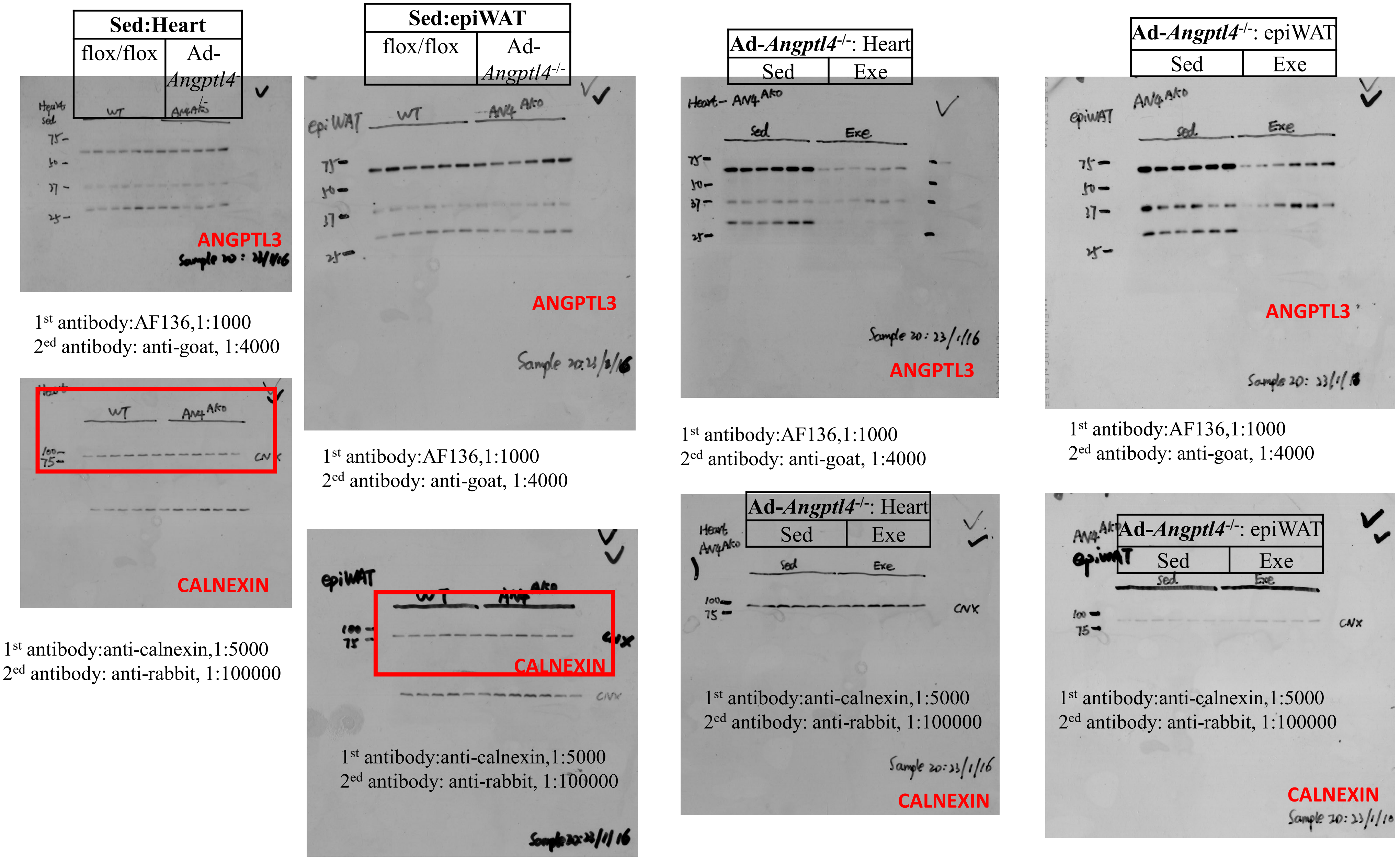

Sed:Heart
flox/flox
Ad-Angptl4-/-
ANGPTL3
1st antibody:AF136,1:1000
2ed antibody: anti-goat, 1:4000
CALNEXIN
1st antibody:anti-calnexin,1:5000
2ed antibody: anti-rabbit, 1:100000
Sed:epiWAT
flox/flox
Ad-Angptl4-/-
ANGPTL3
1st antibody:AF136,1:1000
2ed antibody: anti-goat, 1:4000
CALNEXIN
1st antibody:anti-calnexin,1:5000
2ed antibody: anti-rabbit, 1:100000
Ad-Angptl4-/-: Heart
Sed
Exe
ANGPTL3
1st antibody:AF136,1:1000
2ed antibody: anti-goat, 1:4000
Ad-Angptl4-/-: Heart
Sed
Exe
1st antibody:anti-calnexin,1:5000
2ed antibody: anti-rabbit, 1:100000
CALNEXIN
Ad-Angptl4-/-: epiWAT
Sed
Exe
ANGPTL3
1st antibody:AF136,1:1000
2ed antibody: anti-goat, 1:4000
Ad-Angptl4-/-: epiWAT
Sed
Exe
1st antibody:anti-calnexin,1:5000
2ed antibody: anti-rabbit, 1:100000
CALNEXIN
